# Biomarkers for neurodegeneration impact cognitive function: a longitudinal 1-year case–control study of patients with bipolar disorder and healthy control individuals

**DOI:** 10.1186/s40345-023-00324-5

**Published:** 2024-01-16

**Authors:** Ulla Knorr, Anja Hviid Simonsen, Henrik Zetterberg, Kaj Blennow, Mira Willkan, Julie Forman, Kamilla Miskowiak, Steen Gregers Hasselbalch, Lars Vedel Kessing

**Affiliations:** 1 Department Frederiksberg, Copenhagen Affective Disorder Research Center (CADIC), Psychiatric Center Copenhagen, Nordre Fasanvej 57-59, 2000 Frederiksberg, Denmark; 2 Danish Dementia Research Center, Rigshospitalet, University of Copenhagen, Copenhagen, Denmark; 3 Institute of Neuroscience and Physiology, The Sahlgrenska Academy at University of Gothenburg, Mölndal, Sweden; 4 Clinical Neurochemistry Laboratory, Sahlgrenska University Hospital, Mölndal, Sweden; 5 Department of Neurodegenerative Disease, University College London, Queen Square, London, UK; 6 UK Dementia Research Institute University College London, London, UK; 7 Hong Kong Center for Neurodegenerative Diseases, Hong Kong, China; 8 Section of Biostatistics, Department of Public Health, University of Copenhagen, Copenhagen, Denmark; 9 Department of Psychology, University of Copenhagen, Copenhagen, Denmark; 10 Department of Clinical Medicine, University of Copenhagen, Copenhagen, Denmark

**Keywords:** Cerebrospinal fluid, Bipolar disorder, Amyloid, Tau, Neurogranin, Cognitive function

## Abstract

**Background:**

Abnormalities in cerebrospinal fluid (CSF)-amyloid-beta (Aβ)42, CSF-Aβ40, CSF-Aβ38, CSF-soluble amyloid precursor proteins α and β, CSF-total-tau, CSF-phosphorylated-tau, CSF-neurofilament light protein (NF-L), CSF-neurogranin, plasma-Aβ42, plasma-Aβ40, plasma-total-tau, plasma-NF-L and, serum-S100B during affective episodes may reflect brain changes that could impact cognitive function in patients with bipolar disorder (BD). The study aimed to investigate the association between these biomarkers indicative of Alzheimer’s disease and those reflecting neurodegeneration alongside their impact on cognitive function in patients with BD and healthy control individuals (HC). The primary hypothesis was that GL and VL would increase with increasing levels of CSF-Aβ42 based on data from T0 and T3 in BD and HC jointly.

**Methods:**

In a prospective, longitudinal case–control study euthymic patients with BD (N = 85) and HC (N = 44) were evaluated with clinical assessment and neuropsychological testing at baseline (T0) and during euthymia after a year (T3). Patients’ affective states were recorded weekly as euthymic, subthreshold level, major depression, or (hypo)mania. If an episode occurred during follow-up, the patient was also assessed in post-episode euthymia. Cognitive performance was measured as a global cognitive score (GL) for four cognitive domains including verbal learning and memory (VL).

**Results:**

Estimated in a linear mixed model GL increased with 0.001 for each increase of 1 pg/ml of CSF-Aβ42 (97.5%, CI 0.00043–0.0018, adjusted-p = 0.0005) while VL increased by 0.00089 (97.5%, CI 0.00015–0.0018, adjusted-p = 0.045) in BD and HC jointly. The association was weak, however stronger in patients with BD compared to HC. Associations between other biomarkers including CSF-neurogranin, and cognitive domains were overall weak, and none remained significant after adjustment for multiple testing.

**Limitations:**

Modest sample size. A complete data set regarding both CSF-AB-42 and cognitive test scores was obtained from merely 61 patients with BD and 38 HC individuals.

**Conclusion:**

CSF-Aβ42 may be associated with cognitive dysfunction in patients with BD and HC individuals. The association appeared to be stronger in BD but with overlapping confidence intervals. Hence it remains uncertain whether the association is a general phenomenon or driven by BD.

**Supplementary Information:**

The online version contains supplementary material available at 10.1186/s40345-023-00324-5.

## Introduction

Bipolar disorder (BD) may for subsets of patients be conceptualized as a progressing disorder with an increased risk of recurrence for every new affective episode, progressive shortening of inter-episode intervals with each recurrence (Kessing and Andersen 2017), cognitive impairment (Bora 2018), decreased life expectancy (McIntyre et al. 2020) and a higher risk of developing dementia as found by our group (Kessing and Andersen 2004), and confirmed in subsequent independent meta-analyses (da Silva et al. 2013; Velosa et al. 2020).

Global or selective cognitive impairments occur in 50–70% of patients with BD during clinical remission (Jensen et al. 2016; Bora and Ozerdem 2017; Russo et al. 2017; Bora 2018; Szmulewicz et al. 2019; Knorr et al. 2020). Cognitive impairments may emerge early, among younger newly diagnosed adults (Kjaerstad et al. 2019) and middle-aged patients with BD (Arts et al. 2008; Cullen et al. 2019), and they are robustly associated with impaired functioning (Jimenez-Lopez et al. 2019). However, a recent long-term study (Sparding et al. 2021), prior meta-analyses (Szmulewicz et al. 2019), and further three recently published longitudinal case–control studies of 320 patients with BD and 161 healthy control (HC) individuals evaluated at baseline and at fixed time points during follow-up (1–5 years) did not support the progression of cognitive impairment in BD (Jimenez-Lopez et al. 2019). In clinical practice, patients at increased risk of relapse often present with impaired functioning (Pech et al. 2020). Although the progression of cognitive impairment is not the general rule in BD, patients with BD who have frequent or multiple relapses may constitute a subgroup characterized by slowly progressing neurocognitive impairment not captured in the 1–5-year follow-up studies (Jensen et al. 2016).

Cognitive domains in BD that are most consistently affected in BD include verbal memory and executive functions (Bora 2018; Knorr et al. 2020). Cognitive impairments within these domains resemble the cognitive symptoms associated with Alzheimer’s disease (AD), a neurodegenerative disorder characterized by abnormal accumulation of amyloid-beta (Aβ) peptides in plaques and neurofibrillary tangles in the cerebrum (Obrocki et al. 2020). Core AD biomarkers are low levels of cerebrospinal fluid (CSF)-Aβ42 and high levels of CSF-total tau (t-tau) and hyperphosphorylated tau (p-tau) (Lleo et al. 2019). Aβ42 load even at levels below those seen in typical AD has been associated with neural alterations in younger and middle-aged healthy individuals (Rieck et al. 2015). A previous cross-sectional case–control study found that higher concentrations of CSF-neurofilament light protein (NF-L), ratios of CSF-Aβ42/40 and CSF-Aβ42/38 were consistently associated with altered cognitive performance in euthymic patients with BD whereas CSF-p-tau and CSF-Aβ42 were not (Rolstad et al. 2015).

There is scarce evidence regarding a possible association between the calcium-binding protein S100B and impaired cognition in bipolar disorder (Ottesen et al. 2020).

Neurogranin (NG) is a postsynaptic protein involved in long-term potentiation and synaptic plasticity. NG-CSF levels are significantly higher in AD patients compared to normal controls and mild cognitive impairment (Mavroudis et al. 2020). NF-L may be associated with worse cognition in AD, frontotemporal dementia, and typical cognitive aging (Saunders et al. 2022). NF-L may be more sensitive to subclinical cognitive decline compared to other proposed biomarkers for neurodegeneration (Merluzzi et al. 2019), but studies are needed regarding a possible association between cognitive deficits in BD and NF-L.

The relation between CSF biomarker levels of AD and neurodegeneration, current symptom severity, relapse of mood episodes, and the extent of cognitive impairment in BD is poorly understood (Van Rheenen et al. 2019). Establishing biological correlates of cognitive deterioration might lead to the identification of predictors for the trajectory of cognitive function in BD and as outcomes in clinical trials for the treatment of cognitive impairment in BD.

We, therefore, lack prospective longitudinal case–control studies with intense follow-up to elucidate the direct effect of a relapse of an affective episode on cognitive function in patients with BD. This investigation serves as a subsidiary component of a broader study. We here present the first-ever study on longitudinal associations between cognition and CSF biomarkers of neurodegeneration in patients with BD and healthy control individuals (HC). We have previously from the same cohorts found that abnormalities of amyloid production/clearance during an acute BD episode and that these abnormalities mimic the pattern seen in AD namely decreasing CSF-Aβ42 suggesting an association with brain amyloidosis (Knorr et al. 2022).

### The aims of the study

We aimed at investigating the association between biomarkers indicative of AD and those reflecting neurodegeneration and cognitive function. We aimed to accomplish this by prospectively evaluating associations between biomarkers indicative of AD and neurodegeneration, AND cognitive function in patients with BD and HC individuals. We present a prospective, longitudinal study with repeated measures of cognitive function and both CSF biomarkers and blood biomarkers indicative of AD and those reflecting neurodegeneration during initial euthymia (T0), during post-episode euthymia if an episode occurred (T2), and after a 1-year follow-up (T3) in patients with BD, and HC individuals.

#### Hypotheses

Our primary hypotheses posed before analyses were:

1. Level of CSF-Aβ42 is associated with global cognition in analysis adjusted for age, gender, and follow-up time in patients with BD and HC individuals jointly.
2. Level of CSF-Aβ42 is associated with verbal memory in analysis adjusted for age, gender, and follow-up time in patients with BD and HC individuals jointly.

Further, we included several explorative analyses to investigate the associations between biomarkers (Aβ40, Aβ38, sAPPα, sAPPβ, t-tau, p-tau, p-tau /t-tau, NF-L and NG in CSF, Aβ42, Aβ40, Aβ42/38, Aβ42/40, t-tau, p-tau, p-tau /t-tau and NF-L in plasma and S100B in serum) and cognitive function (global cognition, verbal memory, psychomotor speed, sustained attention, and executive function) in BD and HC jointly.

## Methods

The ABETA study included adult patients with BD and HC individuals in a prospective, longitudinal case–control study with repeated measurements of biomarkers indicative of AD and neurodegeneration during a 1-year follow-up as detailed in our previous paper (Knorr et al. 2019).

The study included a total of 85 patients with BD aged 18–60 years referred to The Copenhagen Affective Disorder Clinic covering a catchment area of 1.6 million people of all psychiatric centers in the Capital Region of Denmark, and who, at the time of inclusion were in a remitted state. The diagnoses of BD were confirmed according to Schedules for Clinical Assessment in Neuropsychiatry interview (Wing et al. 1990). Remission was defined as scores below 8 on both the Hamilton Depression Rating Scale 17-items (HAMD) and the Young Mania Rating Scale (YMRS) (Bech et al. 1986). Further, 44 healthy, age and gender-matched control individuals were recruited via the Danish Donor Register, Frederiksberg Hospital.

### Biological assessments

The participants fasted overnight before the collection of CSF, blood, and urine samples between 0800- and 1000 h a.m. At all time-points (T0, T1, T2, and T3) the clinical assessments, blood, and CSF sampling from the participants were done on the same or the following day.

Specialists of neurology performed a lumbar puncture to collect CSF samples from patients with BD and HC individuals in the lateral decubitus position. The spinal needle was inserted into the L3/L4 or L4/L5 interspace, and a total volume of 10–12 ml of CSF was collected in polypropylene tubes, and gently inverted to avoid gradient effects. Samples were centrifuged on acquisition at 2000*g* for 10 min at + 4 °C and stored in polypropylene tubes in 250 µl aliquots at − 80 °C pending analysis. A general CSF screen was conducted, including albumin, immunoglobulin G (IgG), IgG index, erythrocytes, white blood cells, glucose, and protein.

Board-certified laboratory technicians collected blood samples that were analyzed at the Clinical Biochemical Laboratory at Rigshospitalet, Denmark, regarding standard biochemical parameters including hematological parameters, blood glucose, C-reactive protein, thyroid hormones, lipid status, ions, metabolites, liver enzymes, and lithium levels.

Patients were followed prospectively for a year with an accurate assessment of mood episodes every week. As expected, 50% of the patients experienced a moderate to severe (HAMD or YMRS > 13 for 2 weeks) affective episode during the follow-up period and these patients have given repeated CSF, plasma, and serum samples. Additionally, CSF, plasma, and serum sampling were repeated following remission (T2) and finally after a year (T3) in both patients with BD and HC.

The research was conducted in accordance with the Helsinki Declaration as revised in 1989. The study was approved by the local ethics committee (H6-2014-006) and The Danish Data Protection Agency (J.nr: 2014-58-0015). The study is reported according to the STROBE Statement.

### Biochemical analyses

All analyses regarding biomarkers for AD and neurodegeneration were performed at the Clinical Neurochemistry Laboratory in Mölndal, Sweden, by experienced and board-certified laboratory technicians who were blinded concerning the clinical information. For details, please refer to (Knorr et al. 2022).

The inter-assay coefficient of variability was 8% (sAPPα), 20% (sAPPβ), 2% (Aβ38), 15% (Aβ40), and 13% (Aβ42). The intra-assay coefficient of variability was below 10% for all biomarkers.

### Cognitive function at T0, T2 and T3

Cognitive function was assessed for 1.5 h with a comprehensive neuropsychological test battery as detailed in (Knorr et al. 2020): The validated Danish version of the Screen of Cognitive Impairment in Psychiatry (versions 1, 2, and 3), Rey Auditory Verbal Learning Test (RAVLT) (at T0 and T3: Lists A and B, and at T2: List GeA and Ge-B), Repeatable Battery for the Assessment of Neuropsychological Status; Digit Span (at T0 and T3: Version B and at T2: Version A); Wechsler's Adult Intelligence Scale (3rd edition) digit-letter substitution test; verbal fluency tests with letters S and D (Borkowski et al. 1967); Trail Making Test, part A, and B and, the computerized Cambridge Neuropsychological Test Automated Battery including assessments of memory with the executive function with the Rapid Visual Processing Test (RVIP), as described elsewhere (Purdon 2005). To reduce differences in learning effects we administered the same versions of the RAVLT and RVIP at baseline and follow-up versus another version to the patients that were assessed after an affective episode within the follow-up period. Finally, premorbid verbal intelligence was estimated with the 45-word Danish Adult Reading Task (DART) (Crawford et al. 1987). The sequence of the cognitive tests was the same at each time point. Participants were instructed to be well-rested and avoid caffeine and nicotine on the days of assessment of cognitive function. The participants could request a short intermission pause if needed. Data were complete with a few exceptions. In five separate incidences RVIP data failed due to technical problems and therefore data were disregarded for one HC individual at T3, two patients with BD at T2, and two other patients with BD at T3. The HC participants underwent testing at both T0 and T3, employing identical procedures as those applied to the participants diagnosed with BD.

### Cognitive variables

We created z-scores and composite scores for each time point relative to the healthy norm group performance at baseline by subtracting the healthy mean norm score from the mean patient score and dividing this with the standard deviation for the healthy norm score of each test. TMT and RVIP z-scores were inversed such that lower scores reflect poorer performance consistent with the direction of the other cognition measures. Composite scores for each cognitive domain (‘verbal learning and memory, ‘working memory and executive ‘skills’, ‘sustained attention’ and, ‘psychomotor speed’) at each time point (T0, T2, and T3) were created by averaging the z-scores within the domain. We calculated the global cognitive composite scores by averaging these domain composite scores for each time point.

### Statistical analyses

Data were analyzed according to a preestablished statistical analysis plan. In the primary analyses, the associations of GL and VL with CSF-Aβ42 were evaluated in data from BD and HC jointly, using a linear mixed model including CSF-Aβ42, follow-up time (T0, T3), age, and sex as fixed effects and further assuming an unstructured covariance pattern to account for repeated measurements on each study participant. Results were reported as regression coefficients with 97.5% confidence intervals and Bonferroni-adjusted p-values.

In exploratory analyses, associations between other biomarkers and cognitive functions were evaluated in a similar linear mixed model. The analyses were repeated for the BD and HC sub-groups and with and without adjustment for potential confounders, i.e.

1. Regression coefficient corrected merely for the effect of follow-up time,
2. Corrected for the effect of follow-up time, age, and gender,
3. Corrected for the effect of follow-up time, age, gender, education, smoking, alcohol consumption, and medication (three mood stabilizers lithium (LI), quetiapine (AP), and lamotrigine (AC).

These are referred to as Type 1, Type 2, and Type 3 in figures and tables.

The analyses of the entire data and the HC subgroup were performed with data from T0 and T3. The analyses of the BD subgroup were based on data from T0, T2, and T3. Results were reported as regression coefficients with 95% confidence intervals. P-values were adjusted for multiple testing with the method of Benjamini and Hochberg which controls the false discovery rate (Benjamini et al. 2001).

To visualize the results of the analyses in forest plots, we normalized the regression coefficients by multiplying them with the standard deviation of the biomarker for the HC group at T0. The goodness of fit was evaluated by residual plots. Biomarkers with a markedly skewed distribution were log2-transformed before analysis. Maximum likelihood estimation implicitly handled missing data in the linear mixed models. Extreme outliers were excluded in sensitivity analyses.

All analyses were conducted with SAS software, version 9.4, (Copyright^©^ 2013, SAS Institute Inc., Cary, NC, USA).

This investigation serves as a subsidiary component of a broader study. While a cross-sectional framework would have fulfilled the prerequisites for an examination of associations, our deliberate choice to leverage our expanded dataset has enabled us to fortify the outcomes. Our mixed model integrates data from the cross-section and changes over time, thereby enhancing the statistical power in contrast to isolated analyses (al, 2011). Furthermore, analyses were performed in the joint sample and in subgroup analyses of BD and HC separately to investigate if the associations would be different in the subgroups of BD and HC. The inclusion of data pertaining to individuals without BD provides a platform to discern whether the identified patterns are exclusive to patients with BD or if they exhibit a more generalized presence. The selection of amyloid as the primary outcome aligns with the predetermined statistical analysis plan that underscores primary endpoints.

The choice of the primary cerebrospinal fluid outcome was in line with the previous results regarding this same cohort of patients with BD and HC individuals. Thus, our previous study regarding AD biomarkers (including several biomarkers for neurodegeneration) in bipolar disorder concluded that there were abnormalities of amyloid production/clearance during an acute BD episode. Furthermore, that these abnormalities mimic the pattern seen in AD namely decreasing CSF Aβ42 and may suggest an association with brain amyloidosis (Knorr et al. 2022).

## Results

Results regarding socio-demographic, clinical characteristics, CSF markers, and cognitive function are presented in Table 1 for the 85 patients with BD (N = 49 BDI; N = 37 BDII) and the 44 HC included in the study. The participants contributed data regarding biomarkers and cognitive tests in different degrees, please refer to the flowchart previously presented regarding the cohort (Knorr et al. 2019). Notably, 61 out of 85 patients with BD and 38 out of 44 HC individuals presented both CSF samples and cognitive test score results at baseline T0. Patients with BD and HC individuals were well matched for age and gender and there were no statistically significant differences between the groups regarding years of education. Alcohol consumption was lower, and smoking was more frequent among patients with BD compared to HC (Knorr et al. 2020). Furthermore, results regarding cognitive function are detailed in a prior publication by the authors (Knorr et al. 2020).

**Table 1:** Socio-demographic and clinical characteristics, CSF markers, and cognitive function for patients with bipolar disorder and healthy control individuals at baseline

	Bipolar	Control
N	85	44
Age, median (Q1; Q3)	33 (26; 42)	30.5 (24; 40.5)
Female; N (%)	41 (48.2)	19 (43.2)
Years of education, median (Q1; Q3)	14 (12; 17)	15 (13; 17)
Smokers; N (%)	29 (34.1)	8 (18.2)
Alcohol Consumption, median (Q1; Q3)	0.2 (0; 1)	0.5 (0;1)
Lithium, N (%)	44 (51.8)	0
Antipsychotics, N (%)	35 (41.2)	0
Anticonvulsants, N (%)	42 (49.4)	0
CSF-Aβ42 (pg/ml)	603 (173.91) [25(29.41%)]	631.82 (157.44) [6(13.64%)]
CSF-Aβ40 (pg/ml)	5697.42 (1571.93) [25(29.41%)]	5888.55 (1340.25) [6(13.64%)]
CSF-Aβ38 (pg/ml)	2224 (663.88) [25(29.41%)]	2291.71 (589.32) [6(13.64%)]
CSF-Aβ42/40 ratio	0.11 (0.01) [25(29.41%)]	0.11 (0.01) [6(13.64%)]
CSF-Aβ42/38 ratio	0.27 (0.02) [25(29.41%)]	0.28 (0.02) [6(13.64%)]
CSF-sAPPα (ng/ml)	272.03 (109.68) [25(29.41%)]	295.76 (112.29) [6(13.64%)]
CSF-aAPPβ (ng/ml)	571.32 (191.44) [25(29.41%)]	596.84 (207.44) [6(13.64%)]
CSF-Total Tau (pg/ml)	205.3 (82.05) [25(29.41%)]	195.61 (73.25) [6(13.64%)]
CSF-Phosphyrolated Tau (pg/ml)	33.86 (9.79) [25(29.41%)]	33.21 (8.94) [6(13.64%)]
CSF- Total Tau/phosphorylated Tau^a^	0.17 (0.15; 0.18) [26(30.59%)]	0.17 (0.15; 0.18) [6(13.64%)]
CSF_Neurogranin (pg/ml)^a^	167.5 (139.25; 191.75) [25(29.41%)]	171 (125.75; 216.25) [6(13.64%)]
CSF-Neurofilament Light Chain (pg/ml)^a^	336.5 (246.5; 490.5) [25(29.41%)]	354.5 (214.75; 566.75) [6(13.64%)]
Plasma-Aβ42 (pg/ml)	10.77 (2.45) [4(4.71%)]	10.17 (2.15) [1(2.27%)]
Plasma-Aβ40 (pg/ml)	225.93 (57.87) [3(3.53%)]	215.58 (51.64) [1(2.27%)]
Plasma-Aβ42/40 ratio	0.05 (0.01) [4(4.71%)]	0.05 (0.01) [1(2.27%)]
Plasma-total Tau (pg/ml)	3.03 (0.95) [3(3.53%)]	2.82 (0.88) [1(2.27%)]
Plasma-Neurofilament Light Chain (pg/ml)^a^	6.87 (4.98; 9.11) [5(5.88%)]	5.73 (4.5; 7.84) [1(2.27%)]
Serum-S100 (µg/ml) (pg/ml)^a^	0.04 (0.03; 0.05) [4(4.71%)]	0.04 (0.03; 0.05) [1(2.27%)]
Global cognition	− 0.24 (0.61) [0(0%)]	− 0.01 (0.51) [0(0%)]
Verbal memory	− 0.22 (0.75) [0(0%)]	0 (0.66) [1 (2.27%)]
Executive function	− 0.18 (0.69) [2(2.35%)]	− 0.01 (0.67) [1(2.27%)]
Psychomotor speed	− 0.39 (0.89) [0(0%)]	− 0.02 (0.86) [2(4.55%)]
Sustained attention	− 0.19 (1) [6(7.06%)]	0 (0.91) [1(2.27%)]

^a^Biomarkers are presumed with normal distribution or with log2 normal distribution

The biomarkers assumed to be normally distributed are presented with a mean (sd) [n missing (missing percent)]. The remaining biomarkers are reported with median (Q1; Q3) [n missing (missing percent)]. The cognitive domains are presented with a mean (sd) [n missing (missing percent)]

### Primary analyses

Both global cognition and verbal memory were significantly associated with increasing CSF-Aβ42 in the joint sample of BD and HC (Table 2).

**Table 2:** Associations between CSF Aβ42 and global cognition and verbal memory

Predictor	Outcome	N/observations	Estimate (0.9875 CI)	p-value/adjusted p-value
CSF-Aβ42	Global cognition	99/165	0.0011 $\frac{mL}{pg CSF \mathrm{ A\beta }42}$ (0.00043 $\frac{mL}{pg CSF \mathrm{ A\beta }42}$ – 0.0018 $\frac{mL}{pg CSF \mathrm{ A\beta }42}$)	0.0003/0.0005
CSF-Aβ42	Verbal memory	98/161	0.00089 $\frac{mL}{pg CSF \mathrm{ A\beta }42}$ (0.00015 $\frac{mL}{pg CSF \mathrm{ A\beta }42}$ – 0.0018 $\frac{mL}{pg CSF \mathrm{ A\beta }42}$)	0.0227/0.0454

**Fig. 1:**
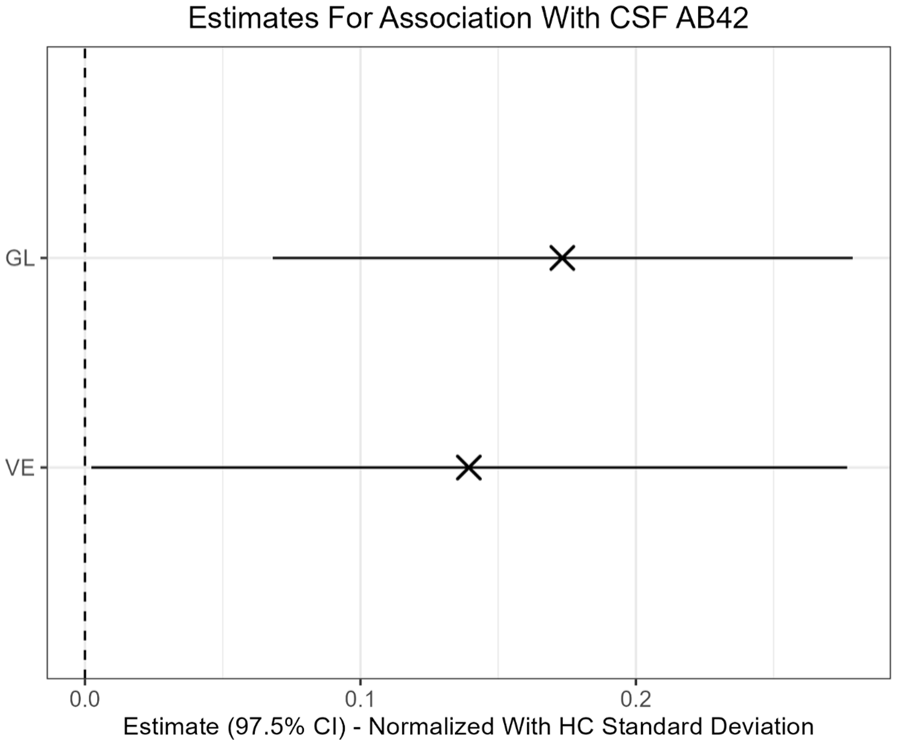
Estimates of associations between cerebrospinal fluid levels of amyloid beta-42 and, global cognition and verbal memory, respectively, in patients with bipolar disorder normalized to healthy control standard deviation. The estimates above are “normalized” so that the estimate in the plot resembles the change in the cognitive score when the biomarker increases with one “healthy control” standard deviation

However, the normalized regression coefficient (Fig. 1) indicates a weak association corresponding to an increase in cognitive Z-score of about 0.1–0.3 for each standard deviation increase in CSF-Aβ42.

### Explorative analyses

**Fig. 2:**
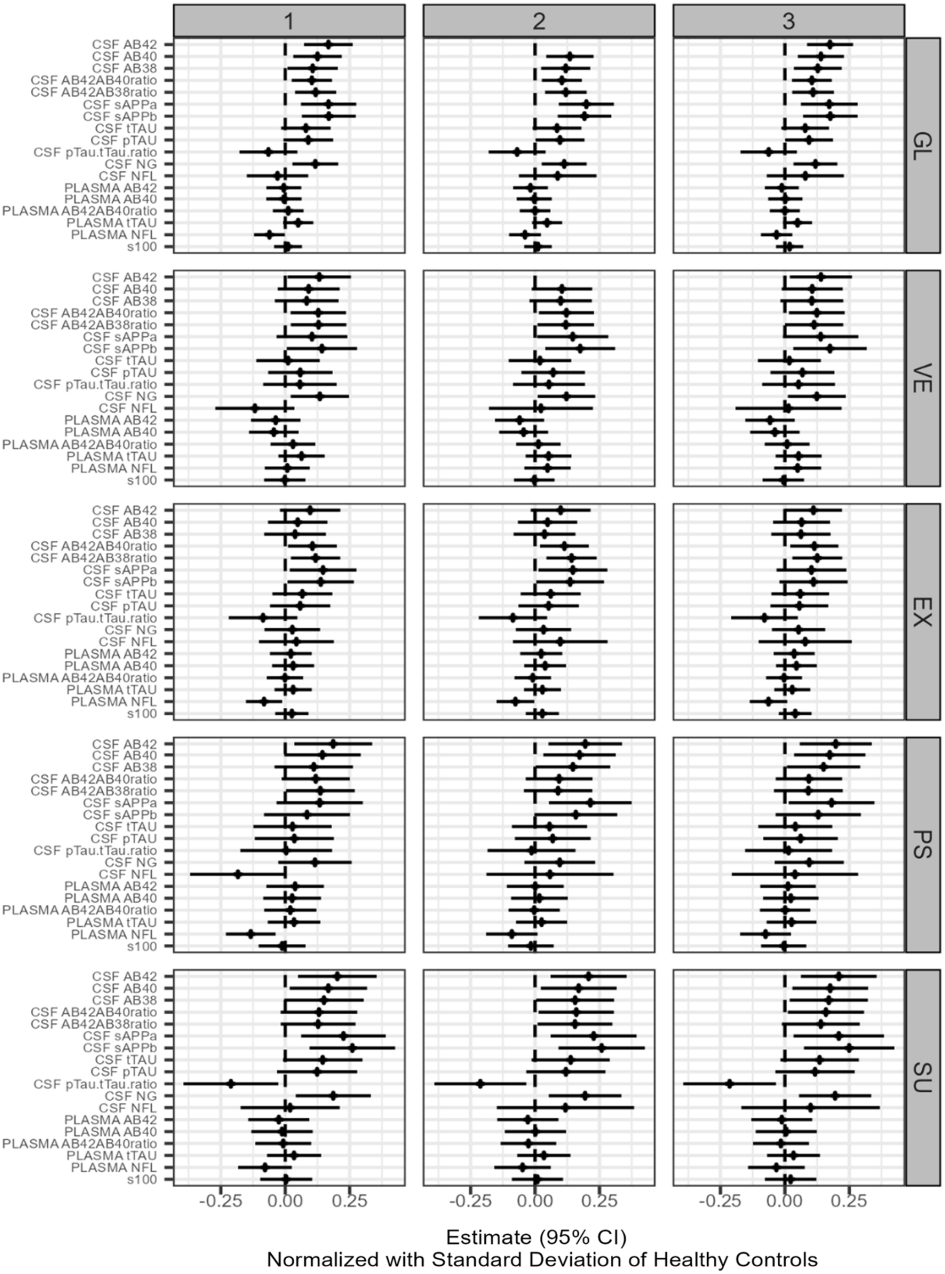
Forest plots of the associations between 18 biomarkers of neurodegeneration versus Global Cognition and the subdomains of Verbal Memory, Executive Function, Psychomotor Speed, and Sustained Attention in all participants were analyzed together (explorative endpoints). The results from the analyses of the effect of a biomarker on cognitive outcome corrected for the effect of (1) follow-up time, (2) follow-up time, age, and gender, and (3) follow-up time, age, gender, education, smoking, alcohol consumption, and for the participants with BP also medication (LI, AP, and AC)

**Fig. 3:**
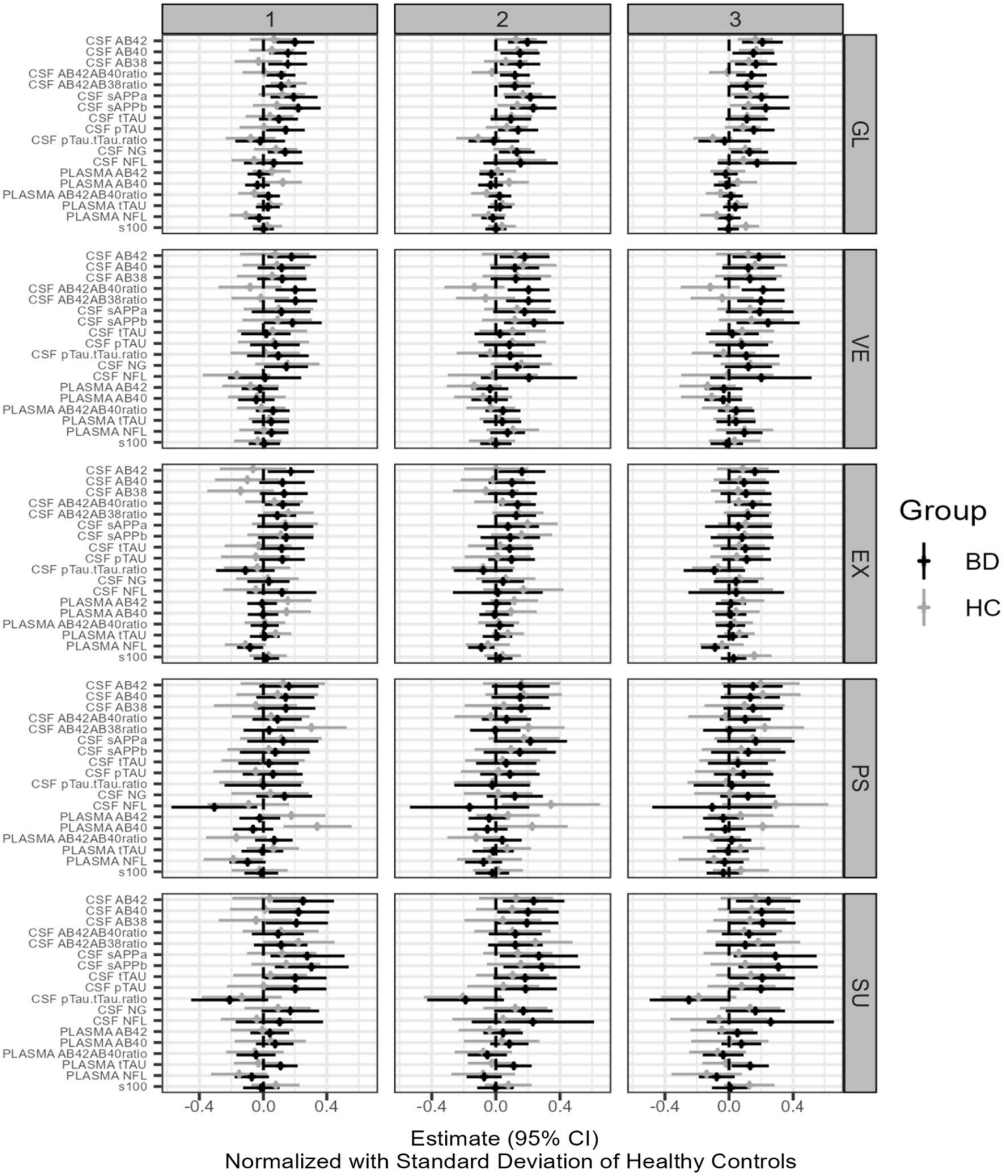
Forest plots of the associations between 18 biomarkers of neurodegeneration versus Global Cognition and the subdomains of Verbal Memory, Executive Function, Psychomotor Speed, and Sustained Attention in patients with bipolar disorder in euthymia and healthy control individuals (explorative endpoints). The results from the analyses of the effect of a biomarker on cognitive outcome corrected for the effect of (1) follow-up time, (2) follow-up time, age, and gender, and (3) follow-up time, age, gender, education, smoking, alcohol consumption, and for the participants with BP also medication (LI, AP, and AC)

The results of the exploratory investigations are displayed in Fig. 2 (all participants together), in Fig. 3 (patients with BD and HC individuals separately), and in Additional file 1: Tables S1–S5. The cognitive function appears to be weakly associated with all the measures CSF-Aβ and CSF-NG both in BD and HC before and after adjustment for potential confounders (age, gender, education, smoking, alcohol consumption, and for the participants with BD also medication). However, none of the associations remained significant after adjustment for multiple testing.

## Discussion

We investigated the associations between levels of biomarkers indicative of AD and neurodegeneration in patients with BD and HC individuals. We used a comprehensive cognitive test battery to reveal important domains of cognitive function. The main finding of this first-ever study on longitudinal associations between CSF biomarkers of neurodegeneration and cognition in patients with BD and HC was that we confirmed our primary hypotheses in predefined analyses showing highly statistically significant associations between CSF-Aβ42 and global cognitive function and verbal memory, respectively. The associations appeared to be stronger in BD but with overlapping confidence intervals. Hence it remains uncertain whether the association is a general phenomenon or driven by BD.

Exploratory analyses did not reveal statistically significant associations after adjustments for multiple testing. However, the results from the forest plots may inspire future investigations when attention is drawn to the related biomarkers regarding amyloidosis (hence, A*β* is a product of sequential cleavage of the membrane glycoprotein APP (amyloid precursor protein) by β- and γ-secretases). Thus, CSF-Aβ40, CSF-Aβ38, CSF-Aβ42/40 ratio, CSF-Aβ42/38 ratio, CSF-sAPPα, and β were associated with global cognitive function and subdomains of cognitive function in several of the unadjusted analyzes. These findings are in line with Rolstad et al. who found that ratios of CSF-Aβ42/40 and CSF-Aβ42/38 were consistently associated with altered cognitive performance in 82 euthymic patients with BD (Rolstad et al. 2015). This finding strengthens the evidence regarding amyloidosis as a possible contributing etiological factor for cognitive impairment in BD. The associations between CSF-Aβ42 and global cognitive function and verbal memory may be indicative of cognitive decline regardless of the BD diagnostic. However, because subjects who experience mental disorders are more likely to develop dementia in older age (Richmond-Rakerd et al. 2022) the rate of these alterations in BD subjects may be higher, and thus, the alterations seen in this present study may add to the understanding of biological underpinnings of an observed increased risk for patients with BD to develop dementia”.

Cognitive function was not associated with tau in our sample. Tau has been associated with cognitive impairment later in the trajectory of Alzheimer’s disease (Pereira et al. 2021) and tauopathy has been suggested in relation to cognitive function in BD and has even been suggested as a novel therapeutic target (Naserkhaki et al. 2019).

Our explorative analyses found an association (when unadjusted for multiple testing) between NG and global cognitions as well as the subdomains of verbal memory and sustained attention, although we cannot rule out that this is a spurious finding due to multiple testing. Recent studies have shown increased NG levels in the CSF of AD patients, and in patients with mild cognitive impairment (MCI). Interestingly, a comparison of stable MCI and MCI that progressed to AD showed significantly higher levels in the CSF of MCI patients who progressed to AD, compared to stable MCI patients (Mavroudis et al. 2020). Thus, neurogranin seems to be a contributing factor in relation to neurodegeneration.

When considering schizophrenia there is evidence of accelerated cognitive decline and increased risk of developing dementia in elderly patients with schizophrenia. However, a recent review concluded that Aβ deposition was not associated with cognitive decline in late-life schizophrenia (Pandolfo et al. 2021). Furthermore, three out of the four studies, which investigated the relationship between Aβ levels and cognitive impairment in schizophrenia, observed no association between the two factors (Pandolfo et al. 2021). These findings may suggest different etiological factors for BD and schizophrenia.

We observed non-significant results regarding S100B. This biomarker has gained much attention as a recent meta-analysis found that S100B was increased in schizophrenia spectrum disorders (24 studies; 1107 patients; standardized mean difference (SMD) = 0.82; 95% confidence interval (CI) = 0.51 to 1.13; I^2^ = 90%), major depression (13 studies; 584 patients; SMD  = 0.57; 95% CI = 0.31 to 0.83; I^2^ = 73%) and bipolar disorder (4 studies; 142 patients; SMD  = 0.55; 95% CI = 0.16 to 0.94; I^2^ = 48%) (Futtrup et al. 2020). However, the findings regarding a correlation between S100B and cognition derived from exploratory analysis in a cross-sectional study may have been without significance (Ottesen et al. 2020). Thus, our data do not support a correlation between S100B and cognitive function.

The main strengths of the study are the highly systematic design, the well-characterized groups of patients and HC including stringent diagnoses, and the intense follow-up by a specialist in psychiatry with weekly assessments of mood states that captured new affective episodes. The patients with BD and HC individuals were well matched. Furthermore, all biochemical analyses were performed in a well-estimated laboratory with the same assay lots and technicians with experience in handling CSF and blood biomarkers of neurodegeneration. Additionally, the internal validity of the biomarkers was high since all biomarkers were stable during the 1-year follow-up in HC individuals. Statistical analyses were reported concerning the false discovery rate.

The limitations were the modest sample size. Furthermore, with a longer follow-up period, the effect of multiple relapses could have been estimated. The patients were appointed to a specialty mood disorder clinic for the treatment of bipolar disorders and the close follow-up may have prevented more relapses. However, it probably facilitated the high adherence to the study protocol. Furthermore, a full data set regarding both CSF-AB-42 and cognitive test scores were obtained from merely 61 patients with BD and 38 HC individuals.”

The clinical implications of the study are uncertain. As for possible treatment, amyloid-oriented therapies targeting individuals with dementia, or its prodrome mild cognitive impairment (MCI), have not yet been successful therapeutically even though they have been associated with reductions in amyloid (Lyketsos 2020). Meanwhile, CSF-mediated clearing of brain waste products via perivascular pathways, named the glymphatic system, is receiving increasing interest, as it offers unexplored perspectives on understanding neurodegenerative diseases. The glymphatic system is involved in the clearance of metabolic by-products such as Aβ from the brain, and its function is believed to lower the risk of developing some of the most common neurodegenerative diseases (Kylkilahti et al. 2021).

Further research perspectives include investigations of changes in the glymphatic system (Rasmussen et al. 2018) that have been proposed as a potential pathogenetic mechanism by which depression functions as a high-risk factor for AD (Xia et al. 2017). The link may be decreased sleep which are symptoms of both depression and (hypo)-mania in BD (Molano et al. 2017). Much needs to be investigated in the future including changes in the blood–brain barrier during an affective episode and modifiable factors of sleep, alcohol, exercise, and medication that may have an impact on cognitive resilience in patients with BD.

## Conclusion

CSF-Aβ42 may be associated with cognitive dysfunction in patients with BD and HC individuals. The association appeared to be stronger in BD but with overlapping confidence intervals. Hence it remains uncertain whether the association is a general phenomenon or driven by BD. Future studies should investigate the mechanisms behind CSF-Aβ42 as a proposed link between BD and AD.

### Supplementary Information

**Additional file 1: Table S1. **Associations between biomarkers and global cognition in patients with bipolar disorder and healthy control individuals. **Table S2. **Associations between biomarkers and verbal memory in patients with bipolar disorder and healthy control individuals. **Table S3.** Associations between biomarkers and executive function in patients with bipolar disorder and healthy control individuals. **Table S4.** Associations between biomarkers and psychomotor speed in patients with bipolar disorder and healthy control individuals. **Table S5.** Associations between biomarkers and sustained attention in patients with bipolar disorder and healthy control individuals.

## Data Availability

The data supporting the finding of this study are available from the first author within reason.
