# Supplementary material for: Biomarkers for neurodegeneration impact cognitive function: a longitudinal 1-year case–control study of patients with bipolar disorder and healthy control individuals
Source: Int J Bipolar Disord. 2024 Jan 16;12:2. doi: 10.1186/s40345-023-00324-5 (PMC10792136; doi:10.1186/s40345-023-00324-5)
Supplement: Supplementary file 1 — Additional file 1: Table S1. Associations between biomarkers and global cognition in patients with bipolar disorder and healthy control individuals. Table S2. Associations between biomarkers and verbal memory in patients with bipolar disorder and healthy control individuals. Table S3. Associations between biomarkers and executive function in patients with bipolar disorder and healthy control individuals. Table S4. Associations between biomarkers and psychomotor speed in patients with bipolar disorder and healthy control individuals. Table S5. Associations between biomarkers and sustained attention in patients with bipolar disorder and healthy control individuals. [file 40345_2023_324_MOESM1_ESM.docx]

Additional file 1: Tables

Analyses of Associations with Global Cognition (GL)

| **Biomarker** | **Cognition** | **n** | **Type 1 Analysis** | **Type 2 Analysis** | **Type 3 Analysis** |
| --- | --- | --- | --- | --- | --- |
| **Analysis of the entire study group** |  |  |  |  |  |
| CSF AB38 | GL | n= 99 obs= 165 | 0.00018 ( 1.5e-05 - 0.00035 ) , 0.0323 / 0.1938 | 2e-04 ( 4e-05 - 0.00036 ) , 0.0148 / 0.1662 | 0.00022 ( 6e-05 - 0.00037 ) , 0.0072 / 0.1419 |
| CSF AB40 | GL | n= 99 obs= 165 | 9.3e-05 ( 2.3e-05 - 0.00016 ) , 0.0095 / 0.1451 | 1e-04 ( 3.2e-05 - 0.00017 ) , 0.0042 / 0.1277 | 1e-04 ( 3.7e-05 - 0.00017 ) , 0.0025 / 0.1056 |
| CSF AB42 | GL | n= 99 obs= 165 | 0.0011 ( 0.00047 - 0.0017 ) , 6e-04 / 0.1056 |  | 0.0011 ( 0.00055 - 0.0017 ) , 2e-04 / 0.0967 |
| CSF AB42AB38ratio | GL | n= 99 obs= 165 | 5.3 ( 1.7 - 8.9 ) , 0.0041 / 0.1277 | 5.3 ( 1.7 - 9 ) , 0.004 / 0.1277 | 4.9 ( 1.3 - 8.5 ) , 0.0085 / 0.1419 |
| CSF AB42AB40ratio | GL | n= 99 obs= 165 | 11 ( 2.6 - 19 ) , 0.0099 / 0.1464 | 11 ( 2.7 - 19 ) , 0.0089 / 0.1419 | 11 ( 2.8 - 19 ) , 0.0083 / 0.1419 |
| CSF NFL | GL | n= 99 obs= 165 | -0.032 ( -0.16 - 0.096 ) , 0.6192 / 0.8001 | 0.095 ( -0.068 - 0.26 ) , 0.2515 / 0.5119 | 0.086 ( -0.076 - 0.25 ) , 0.2964 / 0.5527 |
| CSF NG | GL | n= 99 obs= 165 | 0.24 ( 0.057 - 0.42 ) , 0.0103 / 0.1483 | 0.23 ( 0.053 - 0.41 ) , 0.0114 / 0.1515 | 0.24 ( 0.069 - 0.42 ) , 0.0066 / 0.1419 |
| CSF pTAU | GL | n= 99 obs= 165 | 0.01 ( -0.00079 - 0.021 ) , 0.0692 / 0.2771 | 0.011 ( 0.00019 - 0.021 ) , 0.0461 / 0.2176 | 0.011 ( 0.00012 - 0.021 ) , 0.0475 / 0.2183 |
| CSF pTau.tTau.ratio | GL | n= 99 obs= 163 | -0.22 ( -0.6 - 0.16 ) , 0.2543 / 0.515 | -0.24 ( -0.61 - 0.14 ) , 0.2142 / 0.4664 | -0.21 ( -0.59 - 0.16 ) , 0.2578 / 0.5204 |
| CSF sAPPa | GL | n= 99 obs= 165 | 0.0015 ( 0.00054 - 0.0025 ) , 0.0024 / 0.1056 | 0.0018 ( 0.00082 - 0.0027 ) , 3e-04 / 0.0967 | 0.0015 ( 0.00055 - 0.0025 ) , 0.0024 / 0.1056 |
| CSF sAPPb | GL | n= 99 obs= 165 | 0.00082 ( 0.00031 - 0.0013 ) , 0.0017 / 0.1056 | 0.00092 ( 0.00042 - 0.0014 ) , 4e-04 / 0.0967 | 0.00085 ( 0.00034 - 0.0014 ) , 0.0012 / 0.1056 |
| CSF tTAU | GL | n= 99 obs= 165 | 0.0011 ( -0.00021 - 0.0024 ) , 0.1006 / 0.3247 | 0.0012 ( -0.00013 - 0.0025 ) , 0.0766 / 0.2922 | 0.0011 ( -0.00019 - 0.0023 ) , 0.0945 / 0.3166 |
| PLASMA AB40 | GL | n= 129 obs= 232 | -1e-04 ( -0.0014 - 0.0012 ) , 0.8816 / 0.9435 | -6.2e-05 ( -0.0014 - 0.0013 ) , 0.9265 / 0.9728 | 3.2e-05 ( -0.0013 - 0.0013 ) , 0.9607 / 0.9876 |
| PLASMA AB42 | GL | n= 128 obs= 230 | -0.0026 ( -0.034 - 0.029 ) , 0.8707 / 0.9431 | -0.0083 ( -0.04 - 0.023 ) , 0.6042 / 0.7893 | -0.0055 ( -0.036 - 0.025 ) , 0.7216 / 0.8567 |
| PLASMA AB42AB40ratio | GL | n= 128 obs= 230 | 1.4 ( -5.8 - 8.6 ) , 0.7015 / 0.8513 | 0.00026 ( -7.1 - 7.1 ) , 0.9999 / 1 | -0.026 ( -7 - 7 ) , 0.9942 / 1 |
| PLASMA NFL | GL | n= 128 obs= 228 | -0.1 ( -0.2 - -0.0025 ) , 0.0445 / 0.2156 | -0.065 ( -0.17 - 0.038 ) , 0.2128 / 0.4645 | -0.054 ( -0.16 - 0.048 ) , 0.2964 / 0.5527 |
| PLASMA tTAU | GL | n= 129 obs= 232 | 0.057 ( -0.0098 - 0.12 ) , 0.0941 / 0.3166 | 0.053 ( -0.013 - 0.12 ) , 0.1144 / 0.3472 | 0.056 ( -0.0081 - 0.12 ) , 0.0867 / 0.3054 |
| s100 | GL | n= 129 obs= 232 | 0.02 ( -0.081 - 0.12 ) , 0.6956 / 0.8491 | 0.021 ( -0.079 - 0.12 ) , 0.6807 / 0.8418 | 0.034 ( -0.064 - 0.13 ) , 0.497 / 0.7138 |
| **Analysis of the BD group** |  |  |  |  |  |
| CSF AB38 | GL | n= 61 | 0.00026 ( 5.1e-05 - 0.00047 ) , 0.0153 / 0.1662 | 0.00026 ( 4.1e-05 - 0.00047 ) , 0.02 / 0.1693 | 0.00029 ( 6.3e-05 - 0.00051 ) , 0.0127 / 0.1577 |
| CSF AB40 | GL | n= 61 | 0.00012 ( 2.7e-05 - 2e-04 ) , 0.0112 / 0.1515 | 0.00011 ( 2.1e-05 - 2e-04 ) , 0.0162 / 0.1662 | 0.00011 ( 1.6e-05 - 0.00021 ) , 0.023 / 0.1775 |
| CSF AB42 | GL | n= 61 | 0.0013 ( 0.00051 - 0.002 ) , 0.0013 / 0.1056 | 0.0013 ( 0.00048 - 0.002 ) , 0.0018 / 0.1056 | 0.0013 ( 0.00051 - 0.0021 ) , 0.0016 / 0.1056 |
| CSF AB42AB38ratio | GL | n= 61 | 4.9 ( 0.57 - 9.3 ) , 0.027 / 0.1891 | 5.3 ( 0.79 - 9.8 ) , 0.0218 / 0.1746 | 4.9 ( 0.17 - 9.7 ) , 0.0426 / 0.2156 |
| CSF AB42AB40ratio | GL | n= 61 | 12 ( 2 - 21 ) , 0.0179 / 0.1693 | 12 ( 2.6 - 22 ) , 0.0134 / 0.1625 | 14 ( 4.6 - 24 ) , 0.0046 / 0.1277 |
| CSF NFL | GL | n= 61 | 0.069 ( -0.13 - 0.27 ) , 0.4949 / 0.7132 | 0.17 ( -0.084 - 0.42 ) , 0.1906 / 0.4373 | 0.19 ( -0.077 - 0.46 ) , 0.1607 / 0.408 |
| CSF NG | GL | n= 61 | 0.28 ( 0.053 - 0.5 ) , 0.0161 / 0.1662 | 0.27 ( 0.039 - 0.5 ) , 0.0222 / 0.1746 | 0.26 ( 0.025 - 0.5 ) , 0.0309 / 0.1929 |
| CSF pTAU | GL | n= 61 | 0.016 ( 0.0018 - 0.029 ) , 0.0271 / 0.1891 | 0.016 ( 0.0015 - 0.03 ) , 0.0305 / 0.1929 | 0.017 ( 0.0027 - 0.032 ) , 0.0203 / 0.1693 |
| CSF pTau.tTau.ratio | GL | n= 61 | -0.068 ( -0.6 - 0.46 ) , 0.7988 / 0.9062 | -0.044 ( -0.58 - 0.5 ) , 0.8731 / 0.9431 | -0.096 ( -0.65 - 0.46 ) , 0.7329 / 0.8654 |
| CSF sAPPa | GL | n= 61 | 0.0017 ( 0.00039 - 0.003 ) , 0.0117 / 0.1519 | 0.0019 ( 5e-04 - 0.0033 ) , 0.0086 / 0.1419 | 0.0018 ( 0.00029 - 0.0033 ) , 0.0198 / 0.1693 |
| CSF sAPPb | GL | n= 61 | 0.0011 ( 4e-04 - 0.0017 ) , 0.0021 / 0.1056 | 0.0011 ( 0.00042 - 0.0018 ) , 0.0019 / 0.1056 | 0.0011 ( 0.00038 - 0.0018 ) , 0.0032 / 0.1145 |
| CSF tTAU | GL | n= 61 | 0.0013 ( -0.00038 - 0.003 ) , 0.1267 / 0.3626 | 0.0013 ( -0.00046 - 0.003 ) , 0.1472 / 0.3886 | 0.0015 ( -0.00029 - 0.0033 ) , 0.1001 / 0.3243 |
| PLASMA AB40 | GL | n= 85 | -0.00073 ( -0.0022 - 0.00076 ) , 0.3351 / 0.6005 | -0.00064 ( -0.0021 - 0.00086 ) , 0.3987 / 0.6468 | -3e-04 ( -0.0018 - 0.0012 ) , 0.6951 / 0.8491 |
| PLASMA AB42 | GL | n= 84 | -0.011 ( -0.046 - 0.023 ) , 0.5215 / 0.7266 | -0.013 ( -0.048 - 0.022 ) , 0.4693 / 0.6899 | -0.0099 ( -0.045 - 0.025 ) , 0.5784 / 0.7694 |
| PLASMA AB42AB40ratio | GL | n= 84 | 3.7 ( -5 - 12 ) , 0.4033 / 0.6477 | 2.8 ( -6 - 12 ) , 0.5357 / 0.7355 | 1.4 ( -7.5 - 10 ) , 0.7633 / 0.8897 |
| PLASMA NFL | GL | n= 84 | -0.045 ( -0.16 - 0.074 ) , 0.4582 / 0.6835 | -0.032 ( -0.15 - 0.089 ) , 0.601 / 0.7874 | -0.00095 ( -0.12 - 0.12 ) , 0.9878 / 1 |
| PLASMA tTAU | GL | n= 85 | 0.031 ( -0.053 - 0.12 ) , 0.4649 / 0.6846 | 0.028 ( -0.056 - 0.11 ) , 0.5074 / 0.7192 | 0.044 ( -0.042 - 0.13 ) , 0.3122 / 0.5717 |
| s100 | GL | n= 85 | 0.00095 ( -0.12 - 0.12 ) , 0.9876 / 1 | 0.00085 ( -0.12 - 0.12 ) , 0.989 / 1 | -0.0094 ( -0.13 - 0.11 ) , 0.88 / 0.9435 |
| **Analysis of the HC group** |  |  |  |  |  |
| CSF AB38 | GL | n= 38 | -5.3e-05 ( -0.00031 - 2e-04 ) , 0.6772 / 0.8408 | 1e-04 ( -0.00013 - 0.00033 ) , 0.3686 / 0.6299 | 0.00021 ( 1.1e-05 - 0.00041 ) , 0.0389 / 0.214 |
| CSF AB40 | GL | n= 38 | 4e-05 ( -6.8e-05 - 0.00015 ) , 0.4612 / 0.6846 | 1e-04 ( 4.5e-06 - 2e-04 ) , 0.0405 / 0.2151 | 0.00013 ( 4.6e-05 - 0.00021 ) , 0.0029 / 0.1126 |
| CSF AB42 | GL | n= 38 | 0.00042 ( -0.00053 - 0.0014 ) , 0.3805 / 0.6373 | 8e-04 ( -3.4e-05 - 0.0016 ) , 0.0597 / 0.2494 | 0.001 ( 0.00034 - 0.0017 ) , 0.0044 / 0.1277 |
| CSF AB42AB38ratio | GL | n= 38 | 7.1 ( 2 - 12 ) , 0.0077 / 0.1419 | 5.7 ( 0.61 - 11 ) , 0.0288 / 0.1929 | 5.3 ( 0.18 - 11 ) , 0.0428 / 0.2156 |
| CSF AB42AB40ratio | GL | n= 38 | 0.61 ( -14 - 15 ) , 0.9313 / 0.9734 | -2.6 ( -16 - 10 ) , 0.6944 / 0.8491 | -1 ( -13 - 11 ) , 0.863 / 0.9406 |
| CSF NFL | GL | n= 38 | -0.063 ( -0.22 - 0.091 ) , 0.4163 / 0.6548 | 0.16 ( -0.021 - 0.34 ) , 0.0827 / 0.3028 | 0.097 ( -0.074 - 0.27 ) , 0.2602 / 0.5204 |
| CSF NG | GL | n= 38 | 0.16 ( -0.12 - 0.44 ) , 0.2598 / 0.5204 | 0.21 ( -0.033 - 0.45 ) , 0.0888 / 0.3092 | 0.21 ( -0.0044 - 0.42 ) , 0.0547 / 0.2354 |
| CSF pTAU | GL | n= 38 | 0.00041 ( -0.017 - 0.017 ) , 0.9618 / 0.9876 | 0.0077 ( -0.007 - 0.022 ) , 0.2966 / 0.5527 | 0.0096 ( -0.0031 - 0.022 ) , 0.1341 / 0.3756 |
| CSF pTau.tTau.ratio | GL | n= 38 | -0.27 ( -0.8 - 0.26 ) , 0.3095 / 0.5696 | -0.38 ( -0.84 - 0.087 ) , 0.1092 / 0.3412 | -0.34 ( -0.76 - 0.069 ) , 0.1 / 0.3243 |
| CSF sAPPa | GL | n= 38 | 0.001 ( -0.00028 - 0.0024 ) , 0.1185 / 0.3529 | 0.0015 ( 0.00042 - 0.0026 ) , 0.0075 / 0.1419 | 0.0012 ( 0.00013 - 0.0022 ) , 0.0282 / 0.192 |
| CSF sAPPb | GL | n= 38 | 4e-04 ( -0.00031 - 0.0011 ) , 0.2639 / 0.5222 | 0.00065 ( 5.1e-05 - 0.0012 ) , 0.0342 / 0.2008 | 0.00058 ( 2.5e-05 - 0.0011 ) , 0.0409 / 0.2151 |
| CSF tTAU | GL | n= 38 | 0.00055 ( -0.0015 - 0.0026 ) , 0.594 / 0.7845 | 0.0013 ( -0.00046 - 0.0031 ) , 0.141 / 0.3793 | 0.0015 ( 1.1e-05 - 0.0031 ) , 0.0484 / 0.2199 |
| PLASMA AB40 | GL | n= 44 | 0.0023 ( -5.1e-05 - 0.0047 ) , 0.055 / 0.2354 | 0.0016 ( -0.00079 - 0.004 ) , 0.185 / 0.4357 | 0.001 ( -0.0013 - 0.0034 ) , 0.3835 / 0.6373 |
| PLASMA AB42 | GL | n= 44 | 0.025 ( -0.031 - 0.081 ) , 0.3815 / 0.6373 | 0.0052 ( -0.048 - 0.058 ) , 0.846 / 0.938 | -0.0038 ( -0.054 - 0.047 ) , 0.8814 / 0.9435 |
| PLASMA AB42AB40ratio | GL | n= 44 | -6.8 ( -19 - 5.2 ) , 0.2643 / 0.5222 | -6.9 ( -19 - 4.8 ) , 0.245 / 0.5065 | -6.3 ( -17 - 4.8 ) , 0.2631 / 0.5222 |
| PLASMA NFL | GL | n= 44 | -0.18 ( -0.35 - -0.017 ) , 0.0309 / 0.1929 | -0.075 ( -0.25 - 0.1 ) , 0.3993 / 0.6468 | -0.13 ( -0.3 - 0.048 ) , 0.1511 / 0.3949 |
| PLASMA tTAU | GL | n= 44 | 0.044 ( -0.046 - 0.14 ) , 0.3311 / 0.5952 | 0.046 ( -0.041 - 0.13 ) , 0.2939 / 0.5527 | 0.056 ( -0.026 - 0.14 ) , 0.1777 / 0.4296 |
| s100 | GL | n= 44 | 0.044 ( -0.13 - 0.22 ) , 0.6093 / 0.7935 | 0.07 ( -0.092 - 0.23 ) , 0.3917 / 0.6423 | 0.19 ( 0.041 - 0.35 ) , 0.0142 / 0.1647 |

Analyses of Associations with Verbal Memory (VE)

| **Biomarker** | **Cognition** | **n** | **Type 1 Analysis** | **Type 2 Analysis** | **Type 3 Analysis** |
| --- | --- | --- | --- | --- | --- |
| **Analysis of the entire study group** |  |  |  |  |  |
| CSF AB38 | VE | n= 98 obs= 161 | 0.00014 ( -6.8e-05 - 0.00035 ) , 0.185 / 0.4357 | 0.00017 ( -3.7e-05 - 0.00037 ) , 0.1075 / 0.3397 | 0.00018 ( -3e-05 - 0.00038 ) , 0.0931 / 0.3165 |
| CSF AB40 | VE | n= 98 obs= 161 | 6.8e-05 ( -2.2e-05 - 0.00016 ) , 0.1373 / 0.3768 | 7.8e-05 ( -9.7e-06 - 0.00017 ) , 0.0808 / 0.2988 | 7.9e-05 ( -9.1e-06 - 0.00017 ) , 0.0784 / 0.294 |
| CSF AB42 | VE | n= 98 obs= 161 | 0.00084 ( 6.1e-05 - 0.0016 ) , 0.0349 / 0.202 |  | 0.00089 ( 0.00012 - 0.0017 ) , 0.023 / 0.1775 |
| CSF AB42AB38ratio | VE | n= 98 obs= 161 | 5.8 ( 0.97 - 11 ) , 0.0188 / 0.1693 | 5.3 ( 0.44 - 10 ) , 0.033 / 0.1949 | 5.1 ( 0.022 - 10 ) , 0.049 / 0.2199 |
| CSF AB42AB40ratio | VE | n= 98 obs= 161 | 13 ( 2.2 - 24 ) , 0.0195 / 0.1693 | 13 ( 1.6 - 24 ) , 0.0256 / 0.1847 | 13 ( 1.7 - 24 ) , 0.0247 / 0.1828 |
| CSF NFL | VE | n= 98 obs= 161 | -0.13 ( -0.29 - 0.038 ) , 0.1305 / 0.3722 | 0.025 ( -0.19 - 0.24 ) , 0.8232 / 0.9248 | 0.015 ( -0.21 - 0.24 ) , 0.8912 / 0.9473 |
| CSF NG | VE | n= 98 obs= 161 | 0.27 ( 0.044 - 0.5 ) , 0.0201 / 0.1693 | 0.25 ( 0.021 - 0.48 ) , 0.0323 / 0.1938 | 0.25 ( 0.024 - 0.48 ) , 0.0307 / 0.1929 |
| CSF pTAU | VE | n= 98 obs= 161 | 0.0065 ( -0.0074 - 0.02 ) , 0.3564 / 0.6185 | 0.0079 ( -0.0059 - 0.022 ) , 0.2612 / 0.5207 | 0.0076 ( -0.0063 - 0.021 ) , 0.279 / 0.5394 |
| CSF pTau.tTau.ratio | VE | n= 98 obs= 159 | 0.19 ( -0.29 - 0.68 ) , 0.4296 / 0.6621 | 0.18 ( -0.29 - 0.66 ) , 0.4472 / 0.6771 | 0.18 ( -0.3 - 0.66 ) , 0.4574 / 0.6835 |
| CSF sAPPa | VE | n= 98 obs= 161 | 0.00092 ( -3e-04 - 0.0021 ) , 0.137 / 0.3768 | 0.0013 ( 7.6e-05 - 0.0025 ) , 0.0377 / 0.2104 | 0.0012 ( -6.4e-05 - 0.0025 ) , 0.0622 / 0.2584 |
| CSF sAPPb | VE | n= 98 obs= 161 | 0.00069 ( 3e-05 - 0.0013 ) , 0.0406 / 0.2151 | 0.00084 ( 0.00019 - 0.0015 ) , 0.0118 / 0.1519 | 0.00084 ( 0.00016 - 0.0015 ) , 0.0164 / 0.1662 |
| CSF tTAU | VE | n= 98 obs= 161 | 0.00015 ( -0.0015 - 0.0018 ) , 0.8606 / 0.9406 | 0.00026 ( -0.0014 - 0.0019 ) , 0.7561 / 0.885 | 0.00024 ( -0.0014 - 0.0019 ) , 0.7788 / 0.9001 |
| PLASMA AB40 | VE | n= 128 obs= 225 | -0.00086 ( -0.0027 - 0.001 ) , 0.3654 / 0.6261 | -0.00086 ( -0.0027 - 0.00098 ) , 0.3576 / 0.6185 | -0.00075 ( -0.0026 - 0.0011 ) , 0.4255 / 0.6601 |
| PLASMA AB42 | VE | n= 127 obs= 223 | -0.017 ( -0.062 - 0.027 ) , 0.446 / 0.6765 | -0.028 ( -0.072 - 0.016 ) , 0.2163 / 0.4672 | -0.027 ( -0.071 - 0.018 ) , 0.2371 / 0.495 |
| PLASMA AB42AB40ratio | VE | n= 127 obs= 223 | 3.6 ( -6.8 - 14 ) , 0.4947 / 0.7132 | 1.6 ( -8.8 - 12 ) , 0.7676 / 0.8923 | 1 ( -9.4 - 11 ) , 0.8448 / 0.938 |
| PLASMA NFL | VE | n= 127 obs= 221 | 0.013 ( -0.13 - 0.16 ) , 0.8571 / 0.9395 | 0.081 ( -0.069 - 0.23 ) , 0.2898 / 0.5507 | 0.083 ( -0.07 - 0.24 ) , 0.2847 / 0.5464 |
| PLASMA tTAU | VE | n= 128 obs= 225 | 0.071 ( -0.03 - 0.17 ) , 0.1665 / 0.4163 | 0.059 ( -0.041 - 0.16 ) , 0.2469 / 0.5065 | 0.06 ( -0.041 - 0.16 ) , 0.2403 / 0.5003 |
| s100 | VE | n= 128 obs= 225 | -0.0034 ( -0.15 - 0.15 ) , 0.9642 / 0.9881 | -0.0057 ( -0.15 - 0.14 ) , 0.9387 / 0.9773 | -0.011 ( -0.16 - 0.14 ) , 0.8835 / 0.9435 |
| **Analysis of the BD group** |  |  |  |  |  |
| CSF AB38 | VE | n= 61 | 2e-04 ( -6.6e-05 - 0.00046 ) , 0.1399 / 0.3784 | 0.00021 ( -5.6e-05 - 0.00048 ) , 0.12 / 0.3537 | 0.00022 ( -5.7e-05 - 5e-04 ) , 0.1179 / 0.3527 |
| CSF AB40 | VE | n= 61 | 8.5e-05 ( -2.7e-05 - 2e-04 ) , 0.1366 / 0.3768 | 8.9e-05 ( -2.5e-05 - 2e-04 ) , 0.1234 / 0.3595 | 9e-05 ( -3.2e-05 - 0.00021 ) , 0.145 / 0.385 |
| CSF AB42 | VE | n= 61 | 0.0011 ( 0.00015 - 0.0021 ) , 0.0243 / 0.1827 | 0.0011 ( 0.00015 - 0.0021 ) , 0.0244 / 0.1827 | 0.0012 ( 0.00013 - 0.0022 ) , 0.0274 / 0.19 |
| CSF AB42AB38ratio | VE | n= 61 | 9.2 ( 3.1 - 15 ) , 0.0033 / 0.1145 | 9.1 ( 2.9 - 15 ) , 0.0047 / 0.1277 | 8.9 ( 2.3 - 15 ) , 0.0088 / 0.1419 |
| CSF AB42AB40ratio | VE | n= 61 | 21 ( 7.8 - 34 ) , 0.0022 / 0.1056 | 21 ( 7.7 - 35 ) , 0.0025 / 0.1056 | 22 ( 8.2 - 36 ) , 0.002 / 0.1056 |
| CSF NFL | VE | n= 61 | 0.011 ( -0.24 - 0.26 ) , 0.9308 / 0.9734 | 0.22 ( -0.1 - 0.55 ) , 0.1753 / 0.4264 | 0.22 ( -0.13 - 0.56 ) , 0.2126 / 0.4645 |
| CSF NG | VE | n= 61 | 0.29 ( 0.0084 - 0.58 ) , 0.0438 / 0.2156 | 0.27 ( -0.02 - 0.56 ) , 0.0673 / 0.2713 | 0.25 ( -0.054 - 0.55 ) , 0.1062 / 0.3386 |
| CSF pTAU | VE | n= 61 | 0.0083 ( -0.0093 - 0.026 ) , 0.3512 / 0.6157 | 0.0095 ( -0.0083 - 0.027 ) , 0.2904 / 0.5507 | 0.0089 ( -0.0096 - 0.027 ) , 0.3407 / 0.6066 |
| CSF pTau.tTau.ratio | VE | n= 61 | 0.31 ( -0.35 - 0.98 ) , 0.3467 / 0.6121 | 0.3 ( -0.37 - 0.97 ) , 0.371 / 0.6326 | 0.37 ( -0.34 - 1.1 ) , 0.3017 / 0.5604 |
| CSF sAPPa | VE | n= 61 | 0.001 ( -0.00065 - 0.0026 ) , 0.231 / 0.4886 | 0.0016 ( -0.00015 - 0.0033 ) , 0.0722 / 0.2826 | 0.0017 ( -0.00017 - 0.0036 ) , 0.0747 / 0.2896 |
| CSF sAPPb | VE | n= 61 | 0.00089 ( 1.3e-05 - 0.0018 ) , 0.0468 / 0.2177 | 0.0011 ( 0.00025 - 0.002 ) , 0.0131 / 0.1609 | 0.0012 ( 0.00022 - 0.0021 ) , 0.0162 / 0.1662 |
| CSF tTAU | VE | n= 61 | 0.00024 ( -0.0019 - 0.0024 ) , 0.8204 / 0.9243 | 0.00035 ( -0.0018 - 0.0025 ) , 0.7492 / 0.8795 | 0.00028 ( -0.002 - 0.0025 ) , 0.8039 / 0.9094 |
| PLASMA AB40 | VE | n= 85 | -0.00086 ( -0.0031 - 0.0013 ) , 0.4403 / 0.6704 | -0.00076 ( -0.003 - 0.0014 ) , 0.4979 / 0.7138 | -7e-04 ( -0.003 - 0.0016 ) , 0.5442 / 0.7446 |
| PLASMA AB42 | VE | n= 84 | -0.01 ( -0.063 - 0.043 ) , 0.7084 / 0.8536 | -0.017 ( -0.07 - 0.036 ) , 0.5276 / 0.733 | -0.015 ( -0.07 - 0.039 ) , 0.5807 / 0.7699 |
| PLASMA AB42AB40ratio | VE | n= 84 | 7.2 ( -5.7 - 20 ) , 0.2704 / 0.5278 | 5.3 ( -7.8 - 18 ) , 0.4262 / 0.6601 | 5.3 ( -8.3 - 19 ) , 0.4439 / 0.6746 |
| PLASMA NFL | VE | n= 84 | 0.083 ( -0.095 - 0.26 ) , 0.3581 / 0.6185 | 0.12 ( -0.06 - 0.3 ) , 0.1874 / 0.4359 | 0.16 ( -0.03 - 0.35 ) , 0.0987 / 0.3236 |
| PLASMA tTAU | VE | n= 85 | 0.055 ( -0.079 - 0.19 ) , 0.4209 / 0.6556 | 0.045 ( -0.088 - 0.18 ) , 0.5015 / 0.7157 | 0.049 ( -0.089 - 0.19 ) , 0.4862 / 0.7071 |
| s100 | VE | n= 85 | 0.011 ( -0.17 - 0.19 ) , 0.9082 / 0.9604 | -0.00028 ( -0.18 - 0.18 ) , 0.9975 / 1 | -0.027 ( -0.22 - 0.16 ) , 0.7812 / 0.9001 |
| **Analysis of the HC group** |  |  |  |  |  |
| CSF AB38 | VE | n= 37 | 9.3e-05 ( -0.00028 - 0.00047 ) , 0.6194 / 0.8001 | 0.00022 ( -0.00014 - 0.00058 ) , 0.2259 / 0.484 | 0.00021 ( -0.00015 - 0.00056 ) , 0.2458 / 0.5065 |
| CSF AB40 | VE | n= 37 | 6.3e-05 ( -9.7e-05 - 0.00022 ) , 0.4352 / 0.666 | 0.00013 ( -2.9e-05 - 0.00028 ) , 0.1078 / 0.3397 | 0.00012 ( -2.9e-05 - 0.00027 ) , 0.1107 / 0.3412 |
| CSF AB42 | VE | n= 37 | 0.00047 ( -0.00093 - 0.0019 ) , 0.5059 / 0.7189 | 0.00078 ( -0.00056 - 0.0021 ) , 0.2466 / 0.5065 | 0.00077 ( -0.00052 - 0.0021 ) , 0.236 / 0.4947 |
| CSF AB42AB38ratio | VE | n= 37 | -0.75 ( -8.9 - 7.4 ) , 0.8551 / 0.9385 | -2.9 ( -11 - 5.4 ) , 0.4891 / 0.7099 | -1.9 ( -11 - 6.9 ) , 0.6658 / 0.8361 |
| CSF AB42AB40ratio | VE | n= 37 | -8.6 ( -29 - 12 ) , 0.4032 / 0.6477 | -14 ( -33 - 5.5 ) , 0.1577 / 0.4028 | -12 ( -31 - 6.9 ) , 0.2064 / 0.4568 |
| CSF NFL | VE | n= 37 | -0.18 ( -0.41 - 0.049 ) , 0.1201 / 0.3537 | -0.021 ( -0.32 - 0.28 ) , 0.8908 / 0.9473 | -0.011 ( -0.32 - 0.3 ) , 0.9415 / 0.9781 |
| CSF NG | VE | n= 37 | 0.31 ( -0.11 - 0.73 ) , 0.1402 / 0.3784 | 0.33 ( -0.063 - 0.71 ) , 0.0985 / 0.3236 | 0.27 ( -0.11 - 0.65 ) , 0.1542 / 0.3983 |
| CSF pTAU | VE | n= 37 | 0.0073 ( -0.018 - 0.032 ) , 0.5636 / 0.7605 | 0.011 ( -0.012 - 0.035 ) , 0.3383 / 0.6036 | 0.0083 ( -0.014 - 0.031 ) , 0.4572 / 0.6835 |
| CSF pTau.tTau.ratio | VE | n= 37 | 0.045 ( -0.7 - 0.79 ) , 0.9046 / 0.959 | -0.12 ( -0.83 - 0.59 ) , 0.7355 / 0.8659 | -0.12 ( -0.79 - 0.56 ) , 0.7306 / 0.8639 |
| CSF sAPPa | VE | n= 37 | 0.00085 ( -0.0011 - 0.0028 ) , 0.3845 / 0.6373 | 0.0012 ( -0.00064 - 0.003 ) , 0.1993 / 0.4497 | 0.0012 ( -0.00065 - 0.003 ) , 0.2021 / 0.4522 |
| CSF sAPPb | VE | n= 37 | 0.00043 ( -0.00063 - 0.0015 ) , 0.419 / 0.6552 | 0.00057 ( -0.00042 - 0.0016 ) , 0.2506 / 0.5113 | 0.00068 ( -3e-04 - 0.0017 ) , 0.1697 / 0.4216 |
| CSF tTAU | VE | n= 37 | 0.00079 ( -0.0022 - 0.0038 ) , 0.6018 / 0.7874 | 0.0014 ( -0.0014 - 0.0043 ) , 0.3194 / 0.5796 | 0.0011 ( -0.0016 - 0.0038 ) , 0.407 / 0.6477 |
| PLASMA AB40 | VE | n= 43 | -8e-04 ( -0.0043 - 0.0027 ) , 0.65 / 0.822 | -0.0015 ( -0.0051 - 0.002 ) , 0.3838 / 0.6373 | -0.0021 ( -0.0058 - 0.0016 ) , 0.2593 / 0.5204 |
| PLASMA AB42 | VE | n= 43 | -0.037 ( -0.12 - 0.045 ) , 0.3656 / 0.6261 | -0.063 ( -0.14 - 0.017 ) , 0.1209 / 0.3548 | -0.062 ( -0.14 - 0.02 ) , 0.1338 / 0.3756 |
| PLASMA AB42AB40ratio | VE | n= 43 | -1.7 ( -20 - 17 ) , 0.8549 / 0.9385 | -3.8 ( -22 - 14 ) , 0.6778 / 0.8408 | -1.4 ( -20 - 17 ) , 0.8836 / 0.9435 |
| PLASMA NFL | VE | n= 43 | 0.0054 ( -0.25 - 0.26 ) , 0.9668 / 0.9881 | 0.18 ( -0.097 - 0.45 ) , 0.2016 / 0.4522 | 0.16 ( -0.13 - 0.46 ) , 0.2786 / 0.5394 |
| PLASMA tTAU | VE | n= 43 | 0.042 ( -0.1 - 0.19 ) , 0.5692 / 0.762 | 0.029 ( -0.11 - 0.17 ) , 0.69 / 0.8491 | 0.038 ( -0.11 - 0.19 ) , 0.6092 / 0.7935 |
| s100 | VE | n= 43 | -0.065 ( -0.34 - 0.21 ) , 0.6357 / 0.8167 | -0.045 ( -0.31 - 0.22 ) , 0.7366 / 0.8659 | 0.064 ( -0.23 - 0.36 ) , 0.6705 / 0.8407 |

Analyses of Associations with Executive Function (EX)

| **Biomarker** | **Cognition** | **n** | **Type 1 Analysis** | **Type 2 Analysis** | **Type 3 Analysis** |
| --- | --- | --- | --- | --- | --- |
| **Analysis of the entire study group** |  |  |  |  |  |
| CSF AB38 | EX | n= 97 obs= 158 | 6.5e-05 ( -0.00014 - 0.00027 ) , 0.5295 / 0.7332 | 6.2e-05 ( -0.00014 - 0.00027 ) , 0.5475 / 0.7466 | 0.00011 ( -9e-05 - 3e-04 ) , 0.2865 / 0.5486 |
| CSF AB40 | EX | n= 97 obs= 158 | 3.6e-05 ( -5e-05 - 0.00012 ) , 0.4091 / 0.6485 | 3.6e-05 ( -5e-05 - 0.00012 ) , 0.405 / 0.6477 | 4.8e-05 ( -3.4e-05 - 0.00013 ) , 0.2474 / 0.5065 |
| CSF AB42 | EX | n= 97 obs= 158 | 0.00061 ( -0.00013 - 0.0014 ) , 0.105 / 0.3363 | 0.00063 ( -0.00011 - 0.0014 ) , 0.093 / 0.3165 | 7e-04 ( 2.9e-06 - 0.0014 ) , 0.0491 / 0.2199 |
| CSF AB42AB38ratio | EX | n= 97 obs= 158 | 5.3 ( 0.99 - 9.5 ) , 0.0161 / 0.1662 | 6.3 ( 2 - 11 ) , 0.0047 / 0.1277 | 5.6 ( 1.3 - 9.9 ) , 0.0113 / 0.1515 |
| CSF AB42AB40ratio | EX | n= 97 obs= 158 | 11 ( 1.1 - 21 ) , 0.0293 / 0.1929 | 12 ( 2 - 22 ) , 0.019 / 0.1693 | 12 ( 2.2 - 22 ) , 0.0166 / 0.1663 |
| CSF NFL | EX | n= 97 obs= 158 | 0.047 ( -0.11 - 0.2 ) , 0.5568 / 0.7552 | 0.11 ( -0.093 - 0.3 ) , 0.2953 / 0.5527 | 0.085 ( -0.11 - 0.28 ) , 0.3905 / 0.6423 |
| CSF NG | EX | n= 97 obs= 158 | 0.056 ( -0.16 - 0.28 ) , 0.6142 / 0.7973 | 0.067 ( -0.15 - 0.29 ) , 0.5465 / 0.7465 | 0.11 ( -0.1 - 0.32 ) , 0.3101 / 0.5696 |
| CSF pTAU | EX | n= 97 obs= 158 | 0.0065 ( -0.0066 - 0.02 ) , 0.3284 / 0.5924 | 0.0059 ( -0.0072 - 0.019 ) , 0.3762 / 0.6373 | 0.0063 ( -0.0063 - 0.019 ) , 0.3239 / 0.5856 |
| CSF pTau.tTau.ratio | EX | n= 97 obs= 156 | -0.29 ( -0.75 - 0.16 ) , 0.2038 / 0.4544 | -0.29 ( -0.74 - 0.16 ) , 0.2021 / 0.4522 | -0.27 ( -0.71 - 0.17 ) , 0.228 / 0.486 |
| CSF sAPPa | EX | n= 97 obs= 158 | 0.0013 ( 0.00015 - 0.0025 ) , 0.0266 / 0.189 | 0.0013 ( 0.00012 - 0.0025 ) , 0.0314 / 0.1929 | 0.00092 ( -0.00029 - 0.0021 ) , 0.1342 / 0.3756 |
| CSF sAPPb | EX | n= 97 obs= 158 | 0.00066 ( 4.5e-05 - 0.0013 ) , 0.0357 / 0.2051 | 0.00066 ( 2.2e-05 - 0.0013 ) , 0.0425 / 0.2156 | 0.00054 ( -1e-04 - 0.0012 ) , 0.0984 / 0.3236 |
| CSF tTAU | EX | n= 97 obs= 158 | 0.00091 ( -0.00068 - 0.0025 ) , 0.2616 / 0.5207 | 0.00083 ( -0.00076 - 0.0024 ) , 0.3043 / 0.5627 | 0.00081 ( -0.00071 - 0.0023 ) , 0.293 / 0.5527 |
| PLASMA AB40 | EX | n= 126 obs= 222 | 0.00059 ( -0.00099 - 0.0022 ) , 0.461 / 0.6846 | 0.00076 ( -0.00082 - 0.0023 ) , 0.3448 / 0.6112 | 0.00087 ( -0.00067 - 0.0024 ) , 0.2656 / 0.5234 |
| PLASMA AB42 | EX | n= 125 obs= 220 | 0.01 ( -0.028 - 0.048 ) , 0.5978 / 0.7861 | 0.011 ( -0.027 - 0.049 ) , 0.5652 / 0.7605 | 0.017 ( -0.02 - 0.054 ) , 0.3721 / 0.6332 |
| PLASMA AB42AB40ratio | EX | n= 125 obs= 220 | -0.14 ( -8.6 - 8.3 ) , 0.9739 / 0.9923 | -1.1 ( -9.6 - 7.4 ) , 0.8025 / 0.9092 | -0.4 ( -8.8 - 8 ) , 0.9249 / 0.9728 |
| PLASMA NFL | EX | n= 125 obs= 218 | -0.14 ( -0.26 - -0.02 ) , 0.0221 / 0.1746 | -0.13 ( -0.25 - -0.0048 ) , 0.0417 / 0.2151 | -0.11 ( -0.23 - 0.015 ) , 0.0857 / 0.3044 |
| PLASMA tTAU | EX | n= 126 obs= 222 | 0.035 ( -0.047 - 0.12 ) , 0.4028 / 0.6477 | 0.033 ( -0.049 - 0.11 ) , 0.4299 / 0.6621 | 0.032 ( -0.046 - 0.11 ) , 0.4184 / 0.6552 |
| s100 | EX | n= 126 obs= 222 | 0.048 ( -0.072 - 0.17 ) , 0.4342 / 0.666 | 0.053 ( -0.067 - 0.17 ) , 0.3863 / 0.6373 | 0.074 ( -0.044 - 0.19 ) , 0.215 / 0.4668 |
| **Analysis of the BD group** |  |  |  |  |  |
| CSF AB38 | EX | n= 60 | 0.00022 ( -3.8e-05 - 0.00048 ) , 0.0934 / 0.3165 | 0.00017 ( -8.7e-05 - 0.00043 ) , 0.1878 / 0.4359 | 0.00018 ( -9e-05 - 0.00045 ) , 0.1891 / 0.4373 |
| CSF AB40 | EX | n= 60 | 8.9e-05 ( -1.9e-05 - 2e-04 ) , 0.105 / 0.3363 | 7.5e-05 ( -3.4e-05 - 0.00018 ) , 0.1746 / 0.4264 | 7e-05 ( -4.6e-05 - 0.00019 ) , 0.2363 / 0.4947 |
| CSF AB42 | EX | n= 60 | 0.0011 ( 0.00017 - 0.002 ) , 0.0208 / 0.1705 | 0.001 ( 1e-04 - 0.002 ) , 0.0305 / 0.1929 | 0.001 ( 4.7e-05 - 0.002 ) , 0.0401 / 0.2151 |
| CSF AB42AB38ratio | EX | n= 60 | 3.9 ( -1.6 - 9.3 ) , 0.1624 / 0.4111 | 5.7 ( 0.15 - 11 ) , 0.0442 / 0.2156 | 5.3 ( -0.56 - 11 ) , 0.0753 / 0.2906 |
| CSF AB42AB40ratio | EX | n= 60 | 12 ( 0.84 - 24 ) , 0.036 / 0.2051 | 14 ( 2.4 - 26 ) , 0.0192 / 0.1693 | 15 ( 3.2 - 27 ) , 0.014 / 0.1646 |
| CSF NFL | EX | n= 60 | 0.13 ( -0.11 - 0.36 ) , 0.2968 / 0.5527 | 0.013 ( -0.29 - 0.32 ) , 0.9333 / 0.9742 | 0.048 ( -0.27 - 0.37 ) , 0.7679 / 0.8923 |
| CSF NG | EX | n= 60 | 0.069 ( -0.2 - 0.34 ) , 0.6177 / 0.8001 | 0.091 ( -0.18 - 0.36 ) , 0.5019 / 0.7157 | 0.088 ( -0.19 - 0.37 ) , 0.5346 / 0.7352 |
| CSF pTAU | EX | n= 60 | 0.013 ( -0.003 - 0.029 ) , 0.1077 / 0.3397 | 0.011 ( -0.0054 - 0.027 ) , 0.1877 / 0.4359 | 0.012 ( -0.0047 - 0.029 ) , 0.1535 / 0.3983 |
| CSF pTau.tTau.ratio | EX | n= 60 | -0.38 ( -1 - 0.24 ) , 0.224 / 0.4813 | -0.27 ( -0.89 - 0.36 ) , 0.3986 / 0.6468 | -0.31 ( -0.96 - 0.34 ) , 0.3433 / 0.6098 |
| CSF sAPPa | EX | n= 60 | 0.0012 ( -0.00035 - 0.0028 ) , 0.1244 / 0.36 | 0.00068 ( -0.001 - 0.0024 ) , 0.4356 / 0.666 | 0.00052 ( -0.0013 - 0.0024 ) , 0.5797 / 0.7697 |
| CSF sAPPb | EX | n= 60 | 0.00068 ( -0.00016 - 0.0015 ) , 0.1117 / 0.3413 | 0.00043 ( -0.00046 - 0.0013 ) , 0.3383 / 0.6036 | 0.00039 ( -0.00055 - 0.0013 ) , 0.4121 / 0.6506 |
| CSF tTAU | EX | n= 60 | 0.0016 ( -0.00043 - 0.0035 ) , 0.1232 / 0.3595 | 0.0012 ( -0.00083 - 0.0032 ) , 0.2476 / 0.5065 | 0.0014 ( -0.00071 - 0.0035 ) , 0.1916 / 0.438 |
| PLASMA AB40 | EX | n= 83 | -4.6e-05 ( -0.0019 - 0.0018 ) , 0.9604 / 0.9876 | -0.00017 ( -0.002 - 0.0017 ) , 0.8543 / 0.9385 | 2e-04 ( -0.0017 - 0.0021 ) , 0.8353 / 0.9332 |
| PLASMA AB42 | EX | n= 82 | -0.0037 ( -0.047 - 0.039 ) , 0.8662 / 0.9406 | 0.0016 ( -0.042 - 0.045 ) , 0.9431 / 0.9781 | 0.0053 ( -0.039 - 0.049 ) , 0.8114 / 0.9156 |
| PLASMA AB42AB40ratio | EX | n= 82 | 0.92 ( -9.6 - 11 ) , 0.8623 / 0.9406 | 2.8 ( -7.7 - 13 ) , 0.5955 / 0.7845 | 1.1 ( -9.9 - 12 ) , 0.8431 / 0.938 |
| PLASMA NFL | EX | n= 82 | -0.14 ( -0.28 - -0.0032 ) , 0.0449 / 0.2156 | -0.15 ( -0.29 - -0.014 ) , 0.0311 / 0.1929 | -0.15 ( -0.29 - -0.0015 ) , 0.0477 / 0.2183 |
| PLASMA tTAU | EX | n= 83 | 0.0098 ( -0.095 - 0.11 ) , 0.8544 / 0.9385 | 0.011 ( -0.093 - 0.12 ) , 0.8334 / 0.9332 | 0.026 ( -0.082 - 0.13 ) , 0.6316 / 0.8147 |
| s100 | EX | n= 83 | 0.035 ( -0.11 - 0.18 ) , 0.6381 / 0.8167 | 0.046 ( -0.099 - 0.19 ) , 0.5289 / 0.7332 | 0.053 ( -0.095 - 0.2 ) , 0.4802 / 0.7008 |
| **Analysis of the HC group** |  |  |  |  |  |
| CSF AB38 | EX | n= 37 | -0.00024 ( -0.00059 - 0.00011 ) , 0.174 / 0.4264 | -0.00011 ( -0.00045 - 0.00024 ) , 0.5433 / 0.7446 | 9.6e-05 ( -0.00019 - 0.00038 ) , 0.5052 / 0.7189 |
| CSF AB40 | EX | n= 37 | -7.5e-05 ( -0.00023 - 7.5e-05 ) , 0.3198 / 0.5796 | -1.3e-05 ( -0.00016 - 0.00014 ) , 0.8585 / 0.9398 | 5e-05 ( -7.2e-05 - 0.00017 ) , 0.4101 / 0.6487 |
| CSF AB42 | EX | n= 37 | -0.00041 ( -0.0017 - 0.00091 ) , 0.5345 / 0.7352 | 5.9e-06 ( -0.0013 - 0.0013 ) , 0.9926 / 1 | 0.00054 ( -0.00048 - 0.0016 ) , 0.291 / 0.5507 |
| CSF AB42AB38ratio | EX | n= 37 | 7.1 ( -0.096 - 14 ) , 0.0531 / 0.2323 | 6.2 ( -0.97 - 13 ) , 0.0889 / 0.3092 | 5.1 ( -1.5 - 12 ) , 0.1254 / 0.3601 |
| CSF AB42AB40ratio | EX | n= 37 | 7.1 ( -12 - 26 ) , 0.4565 / 0.6835 | 4.3 ( -14 - 23 ) , 0.635 / 0.8167 | 6.3 ( -9.1 - 22 ) , 0.4156 / 0.6548 |
| CSF NFL | EX | n= 37 | -0.052 ( -0.27 - 0.17 ) , 0.6384 / 0.8167 | 0.19 ( -0.085 - 0.45 ) , 0.1752 / 0.4264 | 0.048 ( -0.2 - 0.3 ) , 0.702 / 0.8513 |
| CSF NG | EX | n= 37 | 0.055 ( -0.35 - 0.46 ) , 0.7839 / 0.9002 | 0.12 ( -0.25 - 0.5 ) , 0.5091 / 0.7196 | 0.13 ( -0.18 - 0.44 ) , 0.3951 / 0.6452 |
| CSF pTAU | EX | n= 37 | -0.0053 ( -0.03 - 0.019 ) , 0.6644 / 0.8356 | 0.0012 ( -0.022 - 0.024 ) , 0.9138 / 0.9651 | 0.0054 ( -0.013 - 0.024 ) , 0.56 / 0.7572 |
| CSF pTau.tTau.ratio | EX | n= 37 | -0.13 ( -0.84 - 0.58 ) , 0.7206 / 0.8567 | -0.27 ( -0.94 - 0.4 ) , 0.42 / 0.6555 | -0.24 ( -0.79 - 0.31 ) , 0.383 / 0.6373 |
| CSF sAPPa | EX | n= 37 | 0.0012 ( -0.00064 - 0.0031 ) , 0.1936 / 0.4405 | 0.0018 ( 8.6e-05 - 0.0034 ) , 0.0398 / 0.2151 | 0.00091 ( -6e-04 - 0.0024 ) , 0.2307 / 0.4886 |
| CSF sAPPb | EX | n= 37 | 0.00052 ( -0.00049 - 0.0015 ) , 0.3026 / 0.561 | 0.00077 ( -0.00015 - 0.0017 ) , 0.0986 / 0.3236 | 0.00046 ( -0.00035 - 0.0013 ) , 0.2583 / 0.5204 |
| CSF tTAU | EX | n= 37 | -0.00042 ( -0.0033 - 0.0025 ) , 0.7714 / 0.8952 | 0.00036 ( -0.0024 - 0.0031 ) , 0.7923 / 0.9042 | 0.00086 ( -0.0013 - 0.0031 ) , 0.4358 / 0.666 |
| PLASMA AB40 | EX | n= 43 | 0.0028 ( -0.00024 - 0.0058 ) , 0.0704 / 0.2782 | 0.0018 ( -0.0013 - 0.0049 ) , 0.2443 / 0.5065 | 0.00083 ( -0.002 - 0.0037 ) , 0.5664 / 0.7608 |
| PLASMA AB42 | EX | n= 43 | 0.072 ( 0.0021 - 0.14 ) , 0.0436 / 0.2156 | 0.053 ( -0.016 - 0.12 ) , 0.1313 / 0.3726 | 0.04 ( -0.023 - 0.1 ) , 0.2113 / 0.464 |
| PLASMA AB42AB40ratio | EX | n= 43 | 1.2 ( -14 - 16 ) , 0.8783 / 0.9435 | 2 ( -13 - 17 ) , 0.7894 / 0.9042 | 3.8 ( -10 - 18 ) , 0.5898 / 0.7806 |
| PLASMA NFL | EX | n= 43 | -0.19 ( -0.4 - 0.026 ) , 0.0836 / 0.3028 | -0.084 ( -0.32 - 0.15 ) , 0.4731 / 0.6916 | -0.074 ( -0.3 - 0.15 ) , 0.5181 / 0.7248 |
| PLASMA tTAU | EX | n= 43 | 0.086 ( -0.029 - 0.2 ) , 0.1398 / 0.3784 | 0.085 ( -0.029 - 0.2 ) , 0.1393 / 0.3784 | 0.073 ( -0.035 - 0.18 ) , 0.1835 / 0.4357 |
| s100 | EX | n= 43 | 0.057 ( -0.16 - 0.27 ) , 0.6007 / 0.7874 | 0.079 ( -0.13 - 0.29 ) , 0.4623 / 0.6846 | 0.29 ( 0.089 - 0.49 ) , 0.0054 / 0.1399 |

Analyses of Associations with Psychomotor Speed (PS)

| **Biomarker** | **Cognition** | **n** | **Type 1 Analysis** | **Type 2 Analysis** | **Type 3 Analysis** |
| --- | --- | --- | --- | --- | --- |
| **Analysis of the entire study group** |  |  |  |  |  |
| CSF AB38 | PS | n= 98 obs= 160 | 0.00019 ( -7.1e-05 - 0.00045 ) , 0.1527 / 0.3977 | 0.00025 ( 4.3e-06 - 0.00049 ) , 0.0461 / 0.2176 | 0.00025 ( 1.3e-05 - 5e-04 ) , 0.0391 / 0.214 |
| CSF AB40 | PS | n= 98 obs= 160 | 0.00011 ( -3e-06 - 0.00022 ) , 0.0565 / 0.2384 | 0.00013 ( 2.4e-05 - 0.00023 ) , 0.0163 / 0.1662 | 0.00013 ( 2.7e-05 - 0.00023 ) , 0.014 / 0.1646 |
| CSF AB42 | PS | n= 98 obs= 160 | 0.0012 ( 0.00022 - 0.0021 ) , 0.0161 / 0.1662 | 0.0012 ( 0.00033 - 0.0021 ) , 0.0078 / 0.1419 | 0.0013 ( 0.00037 - 0.0021 ) , 0.006 / 0.1419 |
| CSF AB42AB38ratio | PS | n= 98 obs= 160 | 6.1 ( 0.14 - 12 ) , 0.045 / 0.2156 | 4 ( -1.9 - 9.9 ) , 0.186 / 0.4359 | 4.1 ( -1.9 - 10 ) , 0.179 / 0.4303 |
| CSF AB42AB40ratio | PS | n= 98 obs= 160 | 12 ( -1.4 - 26 ) , 0.0788 / 0.294 | 9.7 ( -3.8 - 23 ) , 0.1567 / 0.4018 | 9.6 ( -3.7 - 23 ) , 0.1549 / 0.3983 |
| CSF NFL | PS | n= 98 obs= 160 | -0.2 ( -0.4 - 0.0024 ) , 0.0528 / 0.2323 | 0.063 ( -0.2 - 0.33 ) , 0.6433 / 0.8168 | 0.042 ( -0.22 - 0.31 ) , 0.7535 / 0.8833 |
| CSF NG | PS | n= 98 obs= 160 | 0.24 ( -0.055 - 0.53 ) , 0.1116 / 0.3413 | 0.2 ( -0.084 - 0.48 ) , 0.1694 / 0.4216 | 0.19 ( -0.081 - 0.47 ) , 0.1657 / 0.4163 |
| CSF pTAU | PS | n= 98 obs= 160 | 0.0039 ( -0.013 - 0.021 ) , 0.65 / 0.822 | 0.0077 ( -0.0088 - 0.024 ) , 0.3569 / 0.6185 | 0.0068 ( -0.0094 - 0.023 ) , 0.4058 / 0.6477 |
| CSF pTau.tTau.ratio | PS | n= 98 obs= 158 | 0.013 ( -0.59 - 0.62 ) , 0.9674 / 0.9881 | -0.047 ( -0.63 - 0.53 ) , 0.8733 / 0.9431 | 0.049 ( -0.53 - 0.62 ) , 0.8658 / 0.9406 |
| CSF sAPPa | PS | n= 98 obs= 160 | 0.0012 ( -3e-04 - 0.0027 ) , 0.1158 / 0.3493 | 0.0019 ( 0.00048 - 0.0033 ) , 0.0092 / 0.1441 | 0.0016 ( 0.00013 - 0.0031 ) , 0.0328 / 0.1949 |
| CSF sAPPb | PS | n= 98 obs= 160 | 0.00041 ( -4e-04 - 0.0012 ) , 0.3176 / 0.5781 | 0.00076 ( -1.2e-05 - 0.0015 ) , 0.0537 / 0.2327 | 0.00062 ( -0.00017 - 0.0014 ) , 0.1239 / 0.3597 |
| CSF tTAU | PS | n= 98 obs= 160 | 0.00038 ( -0.0017 - 0.0025 ) , 0.7144 / 0.8567 | 0.00077 ( -0.0012 - 0.0028 ) , 0.4481 / 0.6772 | 0.00055 ( -0.0014 - 0.0025 ) , 0.5779 / 0.7694 |
| PLASMA AB40 | PS | n= 128 obs= 225 | 0.00052 ( -0.0017 - 0.0027 ) , 0.6393 / 0.8167 | 0.00033 ( -0.0018 - 0.0025 ) , 0.7607 / 0.8891 | 0.00045 ( -0.0016 - 0.0025 ) , 0.6732 / 0.8408 |
| PLASMA AB42 | PS | n= 127 obs= 223 | 0.018 ( -0.034 - 0.07 ) , 0.4993 / 0.7146 | 0.00033 ( -0.051 - 0.052 ) , 0.99 / 1 | 0.0057 ( -0.044 - 0.056 ) , 0.8236 / 0.9248 |
| PLASMA AB42AB40ratio | PS | n= 127 obs= 223 | 2.4 ( -9.8 - 14 ) , 0.7019 / 0.8513 | -0.4 ( -12 - 12 ) , 0.9477 / 0.9816 | 0.11 ( -12 - 12 ) , 0.9855 / 1 |
| PLASMA NFL | PS | n= 127 obs= 221 | -0.22 ( -0.39 - -0.063 ) , 0.0067 / 0.1419 | -0.15 ( -0.32 - 0.016 ) , 0.0764 / 0.2922 | -0.13 ( -0.29 - 0.039 ) , 0.1349 / 0.3756 |
| PLASMA tTAU | PS | n= 128 obs= 225 | 0.039 ( -0.077 - 0.15 ) , 0.5106 / 0.7198 | 0.029 ( -0.083 - 0.14 ) , 0.6133 / 0.7973 | 0.029 ( -0.08 - 0.14 ) , 0.5957 / 0.7845 |
| s100 | PS | n= 128 obs= 225 | -0.023 ( -0.19 - 0.15 ) , 0.7914 / 0.9042 | -0.03 ( -0.19 - 0.13 ) , 0.7176 / 0.8567 | -0.0083 ( -0.17 - 0.15 ) , 0.9203 / 0.9694 |
| **Analysis of the BD group** |  |  |  |  |  |
| CSF AB38 | PS | n= 61 | 0.00024 ( -8.1e-05 - 0.00056 ) , 0.1417 / 0.3801 | 0.00027 ( -4.1e-05 - 0.00058 ) , 0.0876 / 0.3072 | 0.00025 ( -6e-05 - 0.00057 ) , 0.1108 / 0.3412 |
| CSF AB40 | PS | n= 61 | 1e-04 ( -3.3e-05 - 0.00024 ) , 0.136 / 0.3768 | 0.00011 ( -2e-05 - 0.00025 ) , 0.0948 / 0.3166 | 9.9e-05 ( -3.8e-05 - 0.00024 ) , 0.1544 / 0.3983 |
| CSF AB42 | PS | n= 61 | 0.001 ( -0.00017 - 0.0022 ) , 0.0905 / 0.3107 | 0.00099 ( -0.00016 - 0.0021 ) , 0.0903 / 0.3107 | 0.00095 ( -0.00022 - 0.0021 ) , 0.1097 / 0.3412 |
| CSF AB42AB38ratio | PS | n= 61 | 1.6 ( -5.6 - 8.8 ) , 0.6569 / 0.8288 | -0.18 ( -7.2 - 6.8 ) , 0.9589 / 0.9876 | 0.12 ( -7.2 - 7.5 ) , 0.9737 / 0.9923 |
| CSF AB42AB40ratio | PS | n= 61 | 9 ( -7.1 - 25 ) , 0.2683 / 0.5249 | 6.9 ( -9.1 - 23 ) , 0.3946 / 0.6452 | 10 ( -6 - 27 ) , 0.2109 / 0.464 |
| CSF NFL | PS | n= 61 | -0.33 ( -0.62 - -0.042 ) , 0.0253 / 0.1844 | -0.18 ( -0.58 - 0.23 ) , 0.3843 / 0.6373 | -0.11 ( -0.52 - 0.29 ) , 0.5774 / 0.7694 |
| CSF NG | PS | n= 61 | 0.27 ( -0.092 - 0.63 ) , 0.1426 / 0.3812 | 0.24 ( -0.12 - 0.6 ) , 0.1842 / 0.4357 | 0.24 ( -0.11 - 0.59 ) , 0.1783 / 0.4299 |
| CSF pTAU | PS | n= 61 | 0.0067 ( -0.014 - 0.028 ) , 0.532 / 0.7341 | 0.0098 ( -0.011 - 0.031 ) , 0.3495 / 0.614 | 0.01 ( -0.011 - 0.031 ) , 0.3314 / 0.5952 |
| CSF pTau.tTau.ratio | PS | n= 61 | -0.0038 ( -0.82 - 0.82 ) , 0.9926 / 1 | -0.078 ( -0.88 - 0.73 ) , 0.8477 / 0.938 | 0.06 ( -0.75 - 0.87 ) , 0.8839 / 0.9435 |
| CSF sAPPa | PS | n= 61 | 0.0011 ( -9e-04 - 0.0031 ) , 0.277 / 0.5393 | 0.0019 ( -0.00012 - 0.004 ) , 0.065 / 0.2644 | 0.0015 ( -0.00067 - 0.0036 ) , 0.1728 / 0.4264 |
| CSF sAPPb | PS | n= 61 | 0.00036 ( -0.00071 - 0.0014 ) , 0.5079 / 0.7192 | 0.00072 ( -0.00036 - 0.0018 ) , 0.19 / 0.4373 | 0.00058 ( -0.00054 - 0.0017 ) , 0.3074 / 0.5672 |
| CSF tTAU | PS | n= 61 | 0.00046 ( -0.0021 - 0.0031 ) , 0.7234 / 0.8567 | 0.00088 ( -0.0017 - 0.0035 ) , 0.4953 / 0.7132 | 0.00076 ( -0.0018 - 0.0033 ) , 0.5575 / 0.7552 |
| PLASMA AB40 | PS | n= 85 | -0.0013 ( -0.0037 - 0.0012 ) , 0.3134 / 0.5717 | -0.001 ( -0.0035 - 0.0014 ) , 0.408 / 0.6479 | -0.00047 ( -0.0029 - 0.002 ) , 0.7049 / 0.8522 |
| PLASMA AB42 | PS | n= 84 | -0.011 ( -0.07 - 0.049 ) , 0.7234 / 0.8567 | -0.02 ( -0.079 - 0.04 ) , 0.5156 / 0.7225 | -0.017 ( -0.075 - 0.041 ) , 0.565 / 0.7605 |
| PLASMA AB42AB40ratio | PS | n= 84 | 8.1 ( -6.3 - 22 ) , 0.268 / 0.5249 | 4.8 ( -9.7 - 19 ) , 0.511 / 0.7198 | 1.9 ( -13 - 17 ) , 0.7937 / 0.9042 |
| PLASMA NFL | PS | n= 84 | -0.17 ( -0.35 - 0.024 ) , 0.0861 / 0.3044 | -0.13 ( -0.32 - 0.065 ) , 0.1906 / 0.4373 | -0.046 ( -0.24 - 0.15 ) , 0.6432 / 0.8168 |
| PLASMA tTAU | PS | n= 85 | -0.0075 ( -0.15 - 0.14 ) , 0.92 / 0.9694 | -0.019 ( -0.17 - 0.13 ) , 0.7933 / 0.9042 | -0.0086 ( -0.15 - 0.14 ) , 0.9071 / 0.9604 |
| s100 | PS | n= 85 | -0.025 ( -0.22 - 0.17 ) , 0.798 / 0.9062 | -0.041 ( -0.24 - 0.15 ) , 0.6768 / 0.8408 | -0.067 ( -0.26 - 0.13 ) , 0.4957 / 0.7132 |
| **Analysis of the HC group** |  |  |  |  |  |
| CSF AB38 | PS | n= 37 | -8e-05 ( -0.00052 - 0.00036 ) , 0.7184 / 0.8567 | 8.5e-05 ( -0.00033 - 5e-04 ) , 0.6825 / 0.8427 | 0.00016 ( -0.00026 - 0.00059 ) , 0.4389 / 0.6695 |
| CSF AB40 | PS | n= 37 | 6.6e-05 ( -0.00013 - 0.00026 ) , 0.4917 / 0.7125 | 0.00013 ( -4.9e-05 - 0.00031 ) , 0.1497 / 0.3924 | 0.00016 ( -2.2e-05 - 0.00034 ) , 0.0843 / 0.3028 |
| CSF AB42 | PS | n= 37 | 0.00078 ( -9e-04 - 0.0025 ) , 0.3544 / 0.6185 | 0.001 ( -0.00051 - 0.0026 ) , 0.1846 / 0.4357 | 0.0012 ( -3e-04 - 0.0028 ) , 0.1104 / 0.3412 |
| CSF AB42AB38ratio | PS | n= 37 | 14 ( 3.7 - 23 ) , 0.0079 / 0.1419 | 9.1 ( -1 - 19 ) , 0.0772 / 0.2922 | 10 ( -1.1 - 21 ) , 0.077 / 0.2922 |
| CSF AB42AB40ratio | PS | n= 37 | 4.9 ( -20 - 30 ) , 0.6971 / 0.8491 | -3.3 ( -27 - 20 ) , 0.7809 / 0.9001 | -2.3 ( -26 - 22 ) , 0.8467 / 0.938 |
| CSF NFL | PS | n= 37 | -0.1 ( -0.38 - 0.18 ) , 0.473 / 0.6916 | 0.37 ( 0.04 - 0.7 ) , 0.0288 / 0.1929 | 0.31 ( -0.046 - 0.67 ) , 0.0858 / 0.3044 |
| CSF NG | PS | n= 37 | 0.089 ( -0.41 - 0.59 ) , 0.7193 / 0.8567 | 0.029 ( -0.41 - 0.47 ) , 0.8937 / 0.9487 | 0.011 ( -0.44 - 0.46 ) , 0.962 / 0.9876 |
| CSF pTAU | PS | n= 37 | -0.0055 ( -0.035 - 0.024 ) , 0.7093 / 0.8536 | 0.0019 ( -0.024 - 0.028 ) , 0.8819 / 0.9435 | 0.0029 ( -0.024 - 0.03 ) , 0.8243 / 0.9248 |
| CSF pTau.tTau.ratio | PS | n= 37 | -0.035 ( -0.94 - 0.87 ) , 0.9385 / 0.9773 | -0.074 ( -0.89 - 0.74 ) , 0.8551 / 0.9385 | -0.06 ( -0.88 - 0.76 ) , 0.8841 / 0.9435 |
| CSF sAPPa | PS | n= 37 | 0.00099 ( -0.0013 - 0.0033 ) , 0.3856 / 0.6373 | 0.0016 ( -0.00041 - 0.0035 ) , 0.116 / 0.3493 | 0.0013 ( -0.00082 - 0.0035 ) , 0.2162 / 0.4672 |
| CSF sAPPb | PS | n= 37 | 0.00016 ( -0.0011 - 0.0014 ) , 0.7951 / 0.9046 | 0.00045 ( -0.00064 - 0.0015 ) , 0.4049 / 0.6477 | 0.00037 ( -0.00082 - 0.0015 ) , 0.5318 / 0.7341 |
| CSF tTAU | PS | n= 37 | 8.3e-06 ( -0.0036 - 0.0036 ) , 0.9963 / 1 | 0.00058 ( -0.0026 - 0.0038 ) , 0.7145 / 0.8567 | 0.00073 ( -0.0025 - 0.004 ) , 0.6505 / 0.822 |
| PLASMA AB40 | PS | n= 43 | 0.0066 ( 0.0024 - 0.011 ) , 0.0023 / 0.1056 | 0.0044 ( 0.00011 - 0.0087 ) , 0.0448 / 0.2156 | 0.0041 ( -0.00037 - 0.0085 ) , 0.0718 / 0.2824 |
| PLASMA AB42 | PS | n= 43 | 0.083 ( -0.016 - 0.18 ) , 0.0999 / 0.3243 | 0.035 ( -0.057 - 0.13 ) , 0.4493 / 0.6777 | 0.033 ( -0.062 - 0.13 ) , 0.4862 / 0.7071 |
| PLASMA AB42AB40ratio | PS | n= 43 | -20 ( -43 - 2.4 ) , 0.0783 / 0.294 | -15 ( -36 - 6.7 ) , 0.1748 / 0.4264 | -13 ( -35 - 8.7 ) , 0.2349 / 0.4942 |
| PLASMA NFL | PS | n= 43 | -0.31 ( -0.62 - -0.0047 ) , 0.0467 / 0.2177 | -0.067 ( -0.41 - 0.27 ) , 0.697 / 0.8491 | -0.16 ( -0.53 - 0.21 ) , 0.3959 / 0.6452 |
| PLASMA tTAU | PS | n= 43 | 0.069 ( -0.12 - 0.26 ) , 0.4637 / 0.6846 | 0.079 ( -0.091 - 0.25 ) , 0.3581 / 0.6185 | 0.081 ( -0.09 - 0.25 ) , 0.3469 / 0.6121 |
| s100 | PS | n= 43 | -0.045 ( -0.37 - 0.28 ) , 0.7839 / 0.9002 | 0.025 ( -0.27 - 0.31 ) , 0.8654 / 0.9406 | 0.14 ( -0.19 - 0.46 ) , 0.4 / 0.6468 |

Analyses of Associations with Sustained Attention

| **Biomarker** | **Cognition** | **n** | **Type 1 Analysis** | **Type 2 Analysis** | **Type 3 Analysis** |
| --- | --- | --- | --- | --- | --- |
| **Analysis of the entire study group** |  |  |  |  |  |
| CSF AB38 | SU | n= 97 obs= 158 | 0.00025 ( -7e-06 - 0.00052 ) , 0.0563 / 0.2384 | 0.00026 ( 7.2e-06 - 0.00052 ) , 0.044 / 0.2156 | 0.00029 ( 3.2e-05 - 0.00055 ) , 0.0278 / 0.1909 |
| CSF AB40 | SU | n= 97 obs= 158 | 0.00012 ( 1.3e-05 - 0.00024 ) , 0.0292 / 0.1929 | 0.00013 ( 1.7e-05 - 0.00024 ) , 0.0243 / 0.1827 | 0.00013 ( 2.2e-05 - 0.00024 ) , 0.0194 / 0.1693 |
| CSF AB42 | SU | n= 97 obs= 158 | 0.0013 ( 0.00032 - 0.0023 ) , 0.0098 / 0.1464 | 0.0013 ( 0.00038 - 0.0023 ) , 0.0062 / 0.1419 | 0.0013 ( 4e-04 - 0.0023 ) , 0.0057 / 0.1399 |
| CSF AB42AB38ratio | SU | n= 97 obs= 158 | 5.7 ( -0.77 - 12 ) , 0.0839 / 0.3028 | 6.9 ( 0.44 - 13 ) , 0.0364 / 0.2057 | 6.2 ( -0.52 - 13 ) , 0.0702 / 0.2782 |
| CSF AB42AB40ratio | SU | n= 97 obs= 158 | 14 ( -1.8 - 29 ) , 0.0841 / 0.3028 | 17 ( 1.4 - 32 ) , 0.032 / 0.1938 | 16 ( 1.2 - 32 ) , 0.0349 / 0.202 |
| CSF NFL | SU | n= 97 obs= 158 | 0.02 ( -0.19 - 0.23 ) , 0.8458 / 0.938 | 0.13 ( -0.16 - 0.41 ) , 0.3832 / 0.6373 | 0.11 ( -0.18 - 0.4 ) , 0.4643 / 0.6846 |
| CSF NG | SU | n= 97 obs= 158 | 0.38 ( 0.083 - 0.68 ) , 0.0126 / 0.1577 | 0.4 ( 0.11 - 0.68 ) , 0.0073 / 0.1419 | 0.4 ( 0.11 - 0.68 ) , 0.0068 / 0.1419 |
| CSF pTAU | SU | n= 97 obs= 158 | 0.014 ( -0.0036 - 0.031 ) , 0.118 / 0.3527 | 0.013 ( -0.0038 - 0.031 ) , 0.1254 / 0.3601 | 0.013 ( -0.0041 - 0.03 ) , 0.1349 / 0.3756 |
| CSF pTau.tTau.ratio | SU | n= 97 obs= 156 | -0.72 ( -1.3 - -0.092 ) , 0.0248 / 0.1828 | -0.72 ( -1.3 - -0.12 ) , 0.02 / 0.1693 | -0.73 ( -1.3 - -0.12 ) , 0.0198 / 0.1693 |
| CSF sAPPa | SU | n= 97 obs= 158 | 0.002 ( 0.00055 - 0.0035 ) , 0.0075 / 0.1419 | 0.002 ( 0.00054 - 0.0035 ) , 0.0081 / 0.1419 | 0.0019 ( 3e-04 - 0.0034 ) , 0.02 / 0.1693 |
| CSF sAPPb | SU | n= 97 obs= 158 | 0.0013 ( 0.00046 - 0.0021 ) , 0.0024 / 0.1056 | 0.0012 ( 0.00044 - 0.0021 ) , 0.0028 / 0.1126 | 0.0012 ( 0.00036 - 0.002 ) , 0.0057 / 0.1399 |
| CSF tTAU | SU | n= 97 obs= 158 | 0.002 ( -0.00012 - 0.0041 ) , 0.0645 / 0.2638 | 0.0019 ( -0.00018 - 0.004 ) , 0.0738 / 0.2874 | 0.0018 ( -0.00024 - 0.0039 ) , 0.0822 / 0.3027 |
| PLASMA AB40 | SU | n= 127 obs= 225 | -0.00024 ( -0.0026 - 0.0021 ) , 0.8351 / 0.9332 | 3.3e-05 ( -0.0023 - 0.0023 ) , 0.9777 / 0.9949 | 0.00011 ( -0.0022 - 0.0024 ) , 0.9272 / 0.9728 |
| PLASMA AB42 | SU | n= 126 obs= 223 | -0.012 ( -0.067 - 0.043 ) , 0.679 / 0.841 | -0.013 ( -0.068 - 0.042 ) , 0.6408 / 0.8168 | -0.0055 ( -0.06 - 0.049 ) , 0.8424 / 0.938 |
| PLASMA AB42AB40ratio | SU | n= 126 obs= 223 | -0.94 ( -14 - 12 ) , 0.887 / 0.9454 | -3.1 ( -16 - 9.8 ) , 0.6382 / 0.8167 | -1.8 ( -15 - 11 ) , 0.7808 / 0.9001 |
| PLASMA NFL | SU | n= 126 obs= 221 | -0.13 ( -0.31 - 0.043 ) , 0.1377 / 0.3768 | -0.081 ( -0.26 - 0.1 ) , 0.3826 / 0.6373 | -0.055 ( -0.24 - 0.13 ) , 0.5552 / 0.7552 |
| PLASMA tTAU | SU | n= 127 obs= 225 | 0.039 ( -0.08 - 0.16 ) , 0.522 / 0.7266 | 0.039 ( -0.078 - 0.16 ) , 0.5144 / 0.7225 | 0.038 ( -0.078 - 0.15 ) , 0.5194 / 0.7254 |
| s100 | SU | n= 128 obs= 225 | 0.0069 ( -0.18 - 0.19 ) , 0.942 / 0.9781 | 0.017 ( -0.17 - 0.2 ) , 0.8544 / 0.9385 | 0.041 ( -0.14 - 0.23 ) , 0.6607 / 0.8323 |
| **Analysis of the BD group** |  |  |  |  |  |
| CSF AB38 | SU | n= 60 | 0.00036 ( 2.3e-05 - 0.00069 ) , 0.0366 / 0.2057 | 0.00033 ( -4.8e-06 - 0.00066 ) , 0.0533 / 0.2323 | 0.00035 ( 6.1e-06 - 7e-04 ) , 0.0462 / 0.2176 |
| CSF AB40 | SU | n= 60 | 0.00017 ( 2.4e-05 - 0.00031 ) , 0.0222 / 0.1746 | 0.00015 ( 5.8e-06 - 0.00029 ) , 0.0416 / 0.2151 | 0.00015 ( 2.9e-07 - 3e-04 ) , 0.0496 / 0.2206 |
| CSF AB42 | SU | n= 60 | 0.0016 ( 0.00037 - 0.0028 ) , 0.0114 / 0.1515 | 0.0015 ( 0.00027 - 0.0027 ) , 0.017 / 0.1675 | 0.0016 ( 0.00027 - 0.0029 ) , 0.0181 / 0.1693 |
| CSF AB42AB38ratio | SU | n= 60 | 4.9 ( -2.6 - 12 ) , 0.1993 / 0.4497 | 5.4 ( -2.2 - 13 ) , 0.1591 / 0.4054 | 4.5 ( -3.8 - 13 ) , 0.28 / 0.5396 |
| CSF AB42AB40ratio | SU | n= 60 | 9.5 ( -7.5 - 27 ) , 0.2679 / 0.5249 | 13 ( -4.6 - 30 ) , 0.1482 / 0.3897 | 13 ( -4.6 - 30 ) , 0.1473 / 0.3886 |
| CSF NFL | SU | n= 60 | 0.11 ( -0.19 - 0.41 ) , 0.4626 / 0.6846 | 0.25 ( -0.16 - 0.66 ) , 0.2316 / 0.4886 | 0.28 ( -0.15 - 0.71 ) , 0.1989 / 0.4497 |
| CSF NG | SU | n= 60 | 0.35 ( -0.024 - 0.72 ) , 0.0664 / 0.2691 | 0.35 ( -0.021 - 0.72 ) , 0.0643 / 0.2638 | 0.34 ( -0.041 - 0.71 ) , 0.0799 / 0.297 |
| CSF pTAU | SU | n= 60 | 0.023 ( 0.00089 - 0.045 ) , 0.0416 / 0.2151 | 0.021 ( -0.0012 - 0.043 ) , 0.0631 / 0.2609 | 0.022 ( -0.00051 - 0.045 ) , 0.0552 / 0.2354 |
| CSF pTau.tTau.ratio | SU | n= 60 | -0.72 ( -1.5 - 0.1 ) , 0.0845 / 0.3028 | -0.64 ( -1.5 - 0.17 ) , 0.1199 / 0.3537 | -0.85 ( -1.7 - -0.022 ) , 0.0443 / 0.2156 |
| CSF sAPPa | SU | n= 60 | 0.0025 ( 0.00039 - 0.0045 ) , 0.0205 / 0.1693 | 0.0024 ( 0.00022 - 0.0046 ) , 0.0314 / 0.1929 | 0.0026 ( 0.00026 - 0.0049 ) , 0.0301 / 0.1929 |
| CSF sAPPb | SU | n= 60 | 0.0015 ( 0.00034 - 0.0026 ) , 0.0111 / 0.1515 | 0.0014 ( 0.00023 - 0.0025 ) , 0.0197 / 0.1693 | 0.0015 ( 0.00028 - 0.0027 ) , 0.0161 / 0.1662 |
| CSF tTAU | SU | n= 60 | 0.0028 ( 0.00011 - 0.0054 ) , 0.0412 / 0.2151 | 0.0025 ( -2e-04 - 0.0052 ) , 0.0695 / 0.2771 | 0.0028 ( 3e-05 - 0.0056 ) , 0.0477 / 0.2183 |
| PLASMA AB40 | SU | n= 84 | 0.0014 ( -9e-04 - 0.0037 ) , 0.2292 / 0.4873 | 0.0016 ( -0.00069 - 0.0039 ) , 0.1663 / 0.4163 | 0.0015 ( -0.00092 - 0.0039 ) , 0.2233 / 0.481 |
| PLASMA AB42 | SU | n= 83 | 0.019 ( -0.038 - 0.075 ) , 0.5147 / 0.7225 | 0.021 ( -0.036 - 0.078 ) , 0.4727 / 0.6916 | 0.024 ( -0.034 - 0.082 ) , 0.4068 / 0.6477 |
| PLASMA AB42AB40ratio | SU | n= 83 | -5.5 ( -20 - 9.1 ) , 0.4549 / 0.6835 | -6.4 ( -21 - 8.3 ) , 0.3909 / 0.6423 | -4.4 ( -20 - 11 ) , 0.5683 / 0.762 |
| PLASMA NFL | SU | n= 83 | -0.12 ( -0.3 - 0.057 ) , 0.1808 / 0.4321 | -0.12 ( -0.31 - 0.061 ) , 0.1878 / 0.4359 | -0.13 ( -0.32 - 0.058 ) , 0.1746 / 0.4264 |
| PLASMA tTAU | SU | n= 84 | 0.12 ( -0.0049 - 0.25 ) , 0.0595 / 0.2494 | 0.13 ( 0.00049 - 0.25 ) , 0.0491 / 0.2199 | 0.15 ( 0.018 - 0.28 ) , 0.0258 / 0.1847 |
| s100 | SU | n= 84 | -0.025 ( -0.24 - 0.19 ) , 0.8116 / 0.9156 | -0.0051 ( -0.22 - 0.21 ) , 0.9618 / 0.9876 | 0.018 ( -0.2 - 0.23 ) , 0.8717 / 0.9431 |
| **Analysis of the HC group** |  |  |  |  |  |
| CSF AB38 | SU | n= 37 | -7.9e-05 ( -0.00047 - 0.00031 ) , 0.6873 / 0.8473 | 7.4e-05 ( -0.00033 - 0.00048 ) , 0.7152 / 0.8567 | 0.00022 ( -0.00017 - 0.00061 ) , 0.2521 / 0.5119 |
| CSF AB40 | SU | n= 37 | 7.3e-06 ( -0.00016 - 0.00017 ) , 0.9286 / 0.9731 | 7.6e-05 ( -9.2e-05 - 0.00024 ) , 0.3636 / 0.6261 | 0.00011 ( -5.1e-05 - 0.00026 ) , 0.1802 / 0.4319 |
| CSF AB42 | SU | n= 37 | 0.00025 ( -0.0012 - 0.0017 ) , 0.7364 / 0.8659 | 8e-04 ( -0.00067 - 0.0023 ) , 0.2776 / 0.5393 | 0.0011 ( -0.00033 - 0.0025 ) , 0.1316 / 0.3726 |
| CSF AB42AB38ratio | SU | n= 37 | 10 ( -0.094 - 20 ) , 0.0521 / 0.2306 | 11 ( 0.57 - 21 ) , 0.0391 / 0.214 | 8.1 ( -3.7 - 20 ) , 0.1763 / 0.4277 |
| CSF AB42AB40ratio | SU | n= 37 | 11 ( -13 - 36 ) , 0.3641 / 0.6261 | 11 ( -14 - 35 ) , 0.3855 / 0.6373 | 9.9 ( -14 - 34 ) , 0.4179 / 0.6552 |
| CSF NFL | SU | n= 37 | -0.045 ( -0.29 - 0.2 ) , 0.7077 / 0.8536 | 0.049 ( -0.29 - 0.39 ) , 0.7762 / 0.8994 | -0.068 ( -0.4 - 0.26 ) , 0.6755 / 0.8408 |
| CSF NG | SU | n= 37 | 0.19 ( -0.24 - 0.61 ) , 0.3823 / 0.6373 | 0.25 ( -0.16 - 0.66 ) , 0.2275 / 0.486 | 0.27 ( -0.12 - 0.66 ) , 0.1642 / 0.4145 |
| CSF pTAU | SU | n= 37 | 3.5e-05 ( -0.026 - 0.026 ) , 0.9978 / 1 | 0.0052 ( -0.02 - 0.03 ) , 0.6762 / 0.8408 | 0.0088 ( -0.015 - 0.033 ) , 0.4577 / 0.6835 |
| CSF pTau.tTau.ratio | SU | n= 37 | -0.46 ( -1.3 - 0.39 ) , 0.2805 / 0.5396 | -0.7 ( -1.5 - 0.13 ) , 0.095 / 0.3166 | -0.65 ( -1.4 - 0.15 ) , 0.11 / 0.3412 |
| CSF sAPPa | SU | n= 37 | 0.001 ( -0.00092 - 0.003 ) , 0.2888 / 0.5507 | 0.0012 ( -7e-04 - 0.0031 ) , 0.2042 / 0.4544 | 0.00054 ( -0.0014 - 0.0025 ) , 0.5742 / 0.7675 |
| CSF sAPPb | SU | n= 37 | 0.00069 ( -0.00036 - 0.0017 ) , 0.192 / 0.438 | 0.00075 ( -0.00027 - 0.0018 ) , 0.1445 / 0.385 | 0.00049 ( -0.00056 - 0.0015 ) , 0.3483 / 0.6134 |
| CSF tTAU | SU | n= 37 | 6e-04 ( -0.0026 - 0.0038 ) , 0.7036 / 0.8518 | 0.0014 ( -0.0017 - 0.0045 ) , 0.3573 / 0.6185 | 0.0018 ( -0.0011 - 0.0048 ) , 0.2114 / 0.464 |
| PLASMA AB40 | SU | n= 43 | 0.00086 ( -0.0035 - 0.0052 ) , 0.694 / 0.8491 | 0.00069 ( -0.0039 - 0.0053 ) , 0.7634 / 0.8897 | 9.8e-05 ( -0.0046 - 0.0048 ) , 0.9667 / 0.9881 |
| PLASMA AB42 | SU | n= 43 | -0.0029 ( -0.095 - 0.089 ) , 0.9503 / 0.9831 | -0.018 ( -0.11 - 0.072 ) , 0.6925 / 0.8491 | -0.021 ( -0.11 - 0.07 ) , 0.6419 / 0.8168 |
| PLASMA AB42AB40ratio | SU | n= 43 | -6.4 ( -28 - 15 ) , 0.5567 / 0.7552 | -9.4 ( -31 - 12 ) , 0.3819 / 0.6373 | -8.6 ( -30 - 13 ) , 0.4232 / 0.6579 |
| PLASMA NFL | SU | n= 43 | -0.25 ( -0.55 - 0.041 ) , 0.0899 / 0.3107 | -0.13 ( -0.46 - 0.2 ) , 0.4283 / 0.6621 | -0.23 ( -0.6 - 0.13 ) , 0.2056 / 0.4563 |
| PLASMA tTAU | SU | n= 43 | -0.036 ( -0.21 - 0.14 ) , 0.676 / 0.8408 | -0.03 ( -0.2 - 0.14 ) , 0.7207 / 0.8567 | -0.023 ( -0.19 - 0.14 ) , 0.7846 / 0.9002 |
| s100 | SU | n= 44 | 0.14 ( -0.14 - 0.43 ) , 0.3132 / 0.5717 | 0.14 ( -0.13 - 0.42 ) , 0.2905 / 0.5507 | 0.24 ( -0.057 - 0.53 ) , 0.1121 / 0.3413 |
